# Role of the lipid bilayer in outer membrane protein folding in Gram-negative bacteria

**DOI:** 10.1074/jbc.REV120.011473

**Published:** 2020-06-04

**Authors:** Jim E. Horne, David J. Brockwell, Sheena E. Radford

**Affiliations:** 1 Astbury Centre for Structural Molecular Biology, School of Molecular and Cellular Biology, Faculty of Biological Sciences, University of Leeds, Leeds, United Kingdom; 2 Department of Biochemistry, University of Oxford, Oxford, United Kingdom

**Keywords:** β-barrel assembly machinery (BAM) complex, outer membrane, membrane protein folding, protein-lipid interactions, Gram-negative bacteria, lipid membrane, folding kinetics, OMPome, antibiotic resistance, disorderase, protein folding, membrane bilayer, membrane protein, lipid, BAM complex

## Abstract

β-Barrel outer membrane proteins (OMPs) represent the major proteinaceous component of the outer membrane (OM) of Gram-negative bacteria. These proteins perform key roles in cell structure and morphology, nutrient acquisition, colonization and invasion, and protection against external toxic threats such as antibiotics. To become functional, OMPs must fold and insert into a crowded and asymmetric OM that lacks much freely accessible lipid. This feat is accomplished in the absence of an external energy source and is thought to be driven by the high thermodynamic stability of folded OMPs in the OM. With such a stable fold, the challenge that bacteria face in assembling OMPs into the OM is how to overcome the initial energy barrier of membrane insertion. In this review, we highlight the roles of the lipid environment and the OM in modulating the OMP-folding landscape and discuss the factors that guide folding *in vitro* and *in vivo*. We particularly focus on the composition, architecture, and physical properties of the OM and how an understanding of the folding properties of OMPs *in vitro* can help explain the challenges they encounter during folding *in vivo*. Current models of OMP biogenesis in the cellular environment are still in flux, but the stakes for improving the accuracy of these models are high. OMP folding is an essential process in all Gram-negative bacteria, and considering the looming crisis of widespread microbial drug resistance it is an attractive target. To bring down this vital OMP-supported barrier to antibiotics, we must first understand how bacterial cells build it.

Proteins that span lipid bilayers come in two types, either α-helical or β-barrels. Whereas the cytosolic inner membranes (IMs) of bacteria and the plasma membrane of eukaryotes are comprised only of α-helical membrane proteins, β-barrel outer membrane proteins (OMPs) are found exclusively in the outer membranes (OMs) of diderm bacteria as well as in bacterially derived eukaryotic organelles, such as mitochondria and chloroplasts. The “OMPome” (the complement of OMPs encoded for by a genome) of *Escherichia coli* consists of a large number of proteins ranging in barrel size from 8 to 26 β-strands and includes monomers, small assemblies (dimers, trimers etc.), and oligomeric structures that can form up to 60-stranded pores (Fig. 1). Some OMPs comprise only the integral membrane β-barrel structure, whereas others have soluble domains in the periplasm or on the extracellular surface of the OM. Some OMPs have low copy number or can be absent in the OM under “standard” growth conditions (*e.g.* the *E. coli* porin OmpN) (1–4), and others are present in large copy number (*e.g.* OmpA is estimated to have >100,000 copies in the OM of *E. coli*, whereas OmpX, OmpC, and OmpF are estimated to have >20,000 copies each) (3–7). The functions of OMPs are also very diverse, including passive pores and ion channels (8–11), antibiotic efflux channels (12–15), nutrient uptake systems (16–18), maintenance of structural integrity (19–21), biogenesis and upkeep of the OM (22–26), host cell adhesion and invasion (27–29), biofilm formation (30–33), and cell defense (34, 35). Despite the enormous diversity of OMPs in *E. coli*, it is perhaps surprising that only two are essential: the 16-stranded BamA and 26-stranded LptD (36) (Fig. 1). This is perhaps even more remarkable considering that LptD itself relies on BamA for its assembly (37). LptD's biological role is to insert the lipid component of the outer leaflet of the OM (22, 38). BamA (part of the β-barrel assembly machinery, BAM) is required to fold and insert most (but not all) OMPs into the OM *in vivo* (39) (Table 1). The importance of BAM for the biogenesis of the OM is illustrated by the observation that despite the evolutionary distance between bacteria and eukaryotes, a homologue of BamA, Sam50, is retained in all mitochondria (70). Although only BamA and LptD are essential in *E. coli* under laboratory conditions, it is likely that many more OMPs will be necessary for bacteria to survive, invade new niches, and thrive in diverse environments. Understanding how OMPs fold has been the goal of researchers for approximately the last 3 decades, since the first observations were made that OMPs are capable of folding spontaneously into reconstituted lipid bilayers (71). Initially, the study of the structure and folding mechanisms of OMPs lagged behind those of their α-helical membrane protein counterparts, because the latter are more abundant in eukaryotes and were considered, initially at least, to be more important from the perspective of human health, as half of all approved drugs target α-helical membrane proteins (72, 73). However, in the last 15 years, it has become clear that OMPs are ubiquitous, and some are essential in bacteria (*i.e.* BamA and LptD) or in mitochondria (*i.e.* Sam50 and Tom40) (22, 23, 74–77). Furthermore, the growth in antibiotic-resistant pathogens has highlighted the importance of the OM as a formidable barrier to the entry of antibiotics into bacteria as well as a site of efflux out (78) and as a shield against recognition of surface epitopes by natural or designed antibodies (79–82). Hence, insights gained from studies of OMP folding and biogenesis are also vital for our understanding of human physiology (83) and will be key in guiding our choice of targets for the generation of new antibiotics and vaccines against Gram-negative bacteria (84). Consequently, a number of academic groups and drug companies have ongoing research projects targeting the essential OMPs BamA (the central β-barrel-containing subunit of BAM) and LptD (80, 82, 85–92), with at least six reports of inhibitors of their function in 2018-2019 alone (93–98).

**Figure 1.**
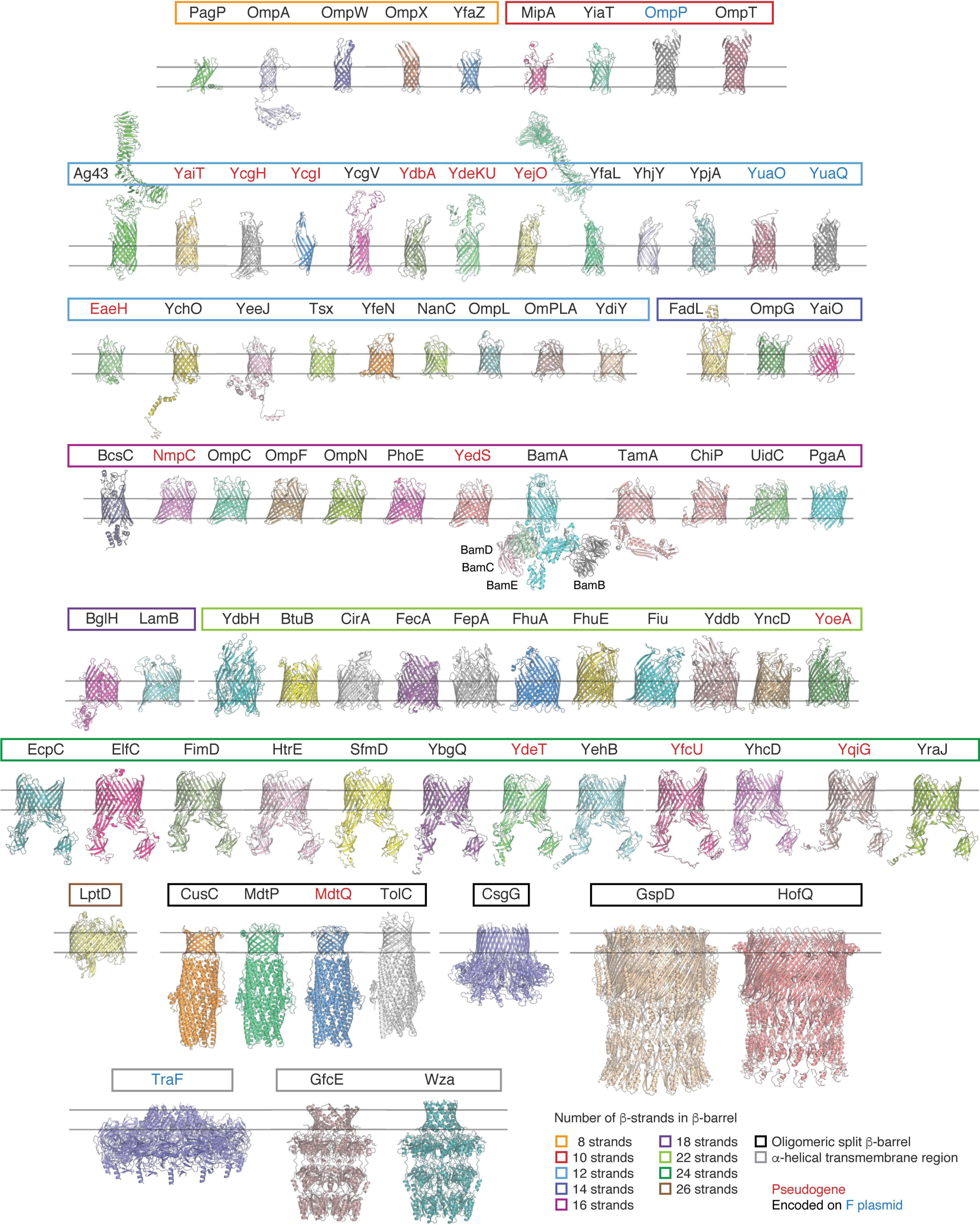
**Structures of transmembrane proteins found in the OM of *E. coli* K-12 MG1655.** A list of all known and predicted transmembrane proteins in the OM of *E. coli* K-12 strain MG1655 was manually curated, creating the “OMP-ome.” The Protein Data Bank was then searched for solved structures of these proteins or close homologues. Where no high-resolution solved 3D structures were available, homology models were generated using the I-TASSER server (RRID:SCR_014627) (396). For two proteins, NfrA (the N4 bacteriophage receptor), and FlgH (the flagellar L-ring protein), no homology models could be generated. Predictions for YaiO, YcgI, YdbH, and YhjY generated deformed or broken barrels (possibly due to a lack of homology to existing structures), but their predictions are displayed to indicate their approximate structure. Extracellular domains of autotransporters have only been shown where accurate models could be built or crystal structures were available. OMPs are grouped here by the number of β-strands and then by protein family. The non-OMP subunits of the BAM complex are labeled *below* the central BamA subunit. Protein names are in *red* if they represent pseudogenes (inactivated by mutation in this strain) and *blue* if they are encoded on the F plasmid. The *color* of the *box* surrounding the protein names represents the number of β-strands in the β-barrel. *Light Orange*, 8; *red*, 10; *light blue*, 12; *violet*, 14; *pink*, 16; *purple*, 18; *light green*, 22; *dark green*, 24; *brown*, 26; *black*, oligomeric split β-barrel; *gray*, α-helical transmembrane region. Structures were aligned with each other by their β-barrel domains and rendered individually in PyMOL 2.X (Schrödinger, LLC). A list of the proteins with their associated family and PDB code can be found in Table S1.

**Table 1:** **Summary of BAM-dependent and BAM-independent OMPs in the OM of different bacteria** Listed are studies that present evidence a link is present (BAM catalysis–involved) or absent (BAM-independent folding) between the biogenesis of a particular OMP and the presence of the BAM complex. This list includes *in vivo* studies and *in vitro* folding studies performed with polar lipid extract from *E. coli.*

OMP(s)	Family	No. of β-strands	Organism	Reference
**BAM catalysis–involved**				
OmpA, OmpX, OmpT, OmPLA, OmpG	*Varied small barrels*	8–14	*E. coli^a^*	Refs. 40–46
*Various*	Autotransporters	12	*E. coli*	Refs. 46–52
OprD	Outer membrane porin	18	*P. aeruginosa*	Ref. 53
LamB	Sugar porin	18	*E. coli*	Refs. 54, 55
*Various*	TonB-dependent receptors	22	*Caulobacter crescentus*	Refs. 56, 57
TolC	Outer membrane factor	3 × 4 (12)	*E. coli*	Ref. 58
FimD*^b^*	Fimbrial usher	24	*E. coli*	Ref. 59
LptD	LPS assembly	26	*E. coli*	Refs. 37, 54, 60–62
PilQ*^b^*	Type IV pilus secretin	14 × 4 (56)	*Neisseria meningitidis*	Refs. 23, 63
**BAM-independent folding**				
PulD*^b^*, XcpQ*^b^*, GspD*^b^*	T2SS secretin	15 × 4 (60)	*Klebsiella oxytoca, P. aeruginosa, E. coli*	Refs. 64–67
pIV*^b^*	Phage secretin	?	Phage f1	Refs. 68, 69
CsgG*^b^*^,^*^c^*	Curli secretion	9 × 4 (36)	*E. coli*	Ref. 67

*^a^* These studies were all performed *in vitro*.

*^b^* These proteins are often assembled as part of larger protein machineries or export/import pathways and may also include their own targeting and assembly factors.

*^c^* Also contains an N-terminal lipid anchor.

This review aims to provide a holistic view of our current understanding of the process of OMP biogenesis, including 1) the composition and physical and chemical properties of the OM *in vivo*; 2) current knowledge of the determinants of OMP folding through *in vitro* studies; and 3) how OMP folding depends on parameters such as the lipid composition, physical environment, and the presence/absence of BAM. Although information is drawn from different organisms, we focus on OMPs and the OM of *E. coli* because of the position of this bacterium as the *de facto* model organism for studying these processes.

## Another brick in the wall: Building the OM

To understand OMP folding and biogenesis, it is first important to review our current understanding of the composition and architecture of the environment in which this process takes place: the complex and crowded bacterial OM.

### Lipid types found in the OM

**Figure 2.**
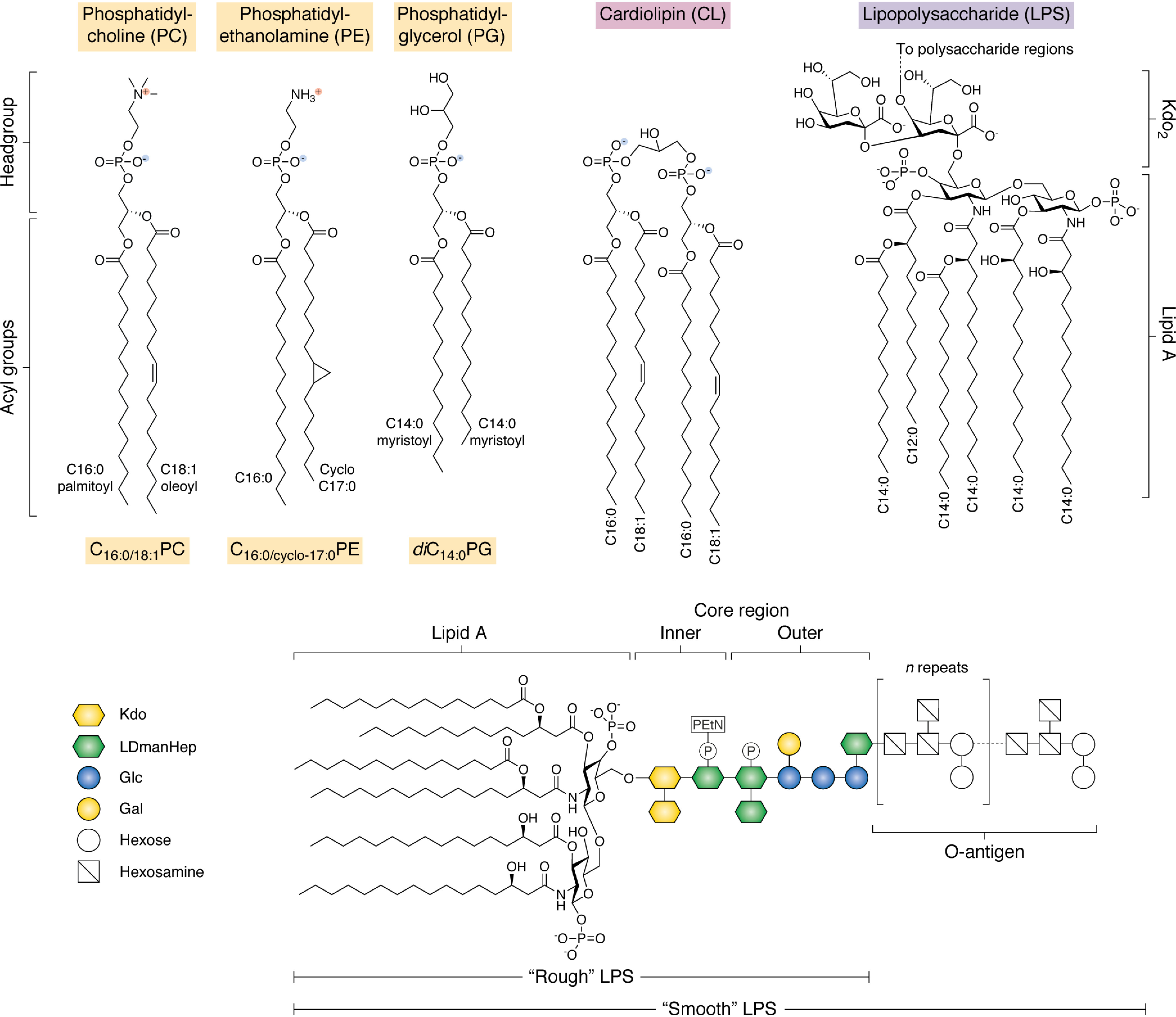
**Common lipid types found in bacterial outer membranes and/or used in *in vitro* studies of OMP folding.** *Top*, schematic of the generic structure of phospholipids and LPS. Bacterial lipids can be conceptualized as having two “domains”: a polar headgroup and a hydrophobic acyl tail region. In phospholipids, the acyl tails are connected by an ester linkage to a phosphate group and a variable headgroup region. PC and PE are zwitterionic, whereas PG carries a net negative charge. Note that PC lipids are not commonly found in bacterial membranes but are often used for OMP folding-studies *in vitro* due to their net neutral charge and propensity to form bilayers. Cardiolipin comprises two acyl tail regions connected by phosphate groups via a glycerol linkage and carries a net double negative charge. LPS is found exclusively in the OM of Gram-negative bacteria and varies considerably between species in both the number and length of acyl tails in the lipid A region and the sugar composition in the polysaccharide region (shown *below*). Here the most common structure of lipid A-Kdo_2_ for *E. coli* K-12 LPS is shown in full. *Bottom*, the architecture of a generic LPS is shown. The lipid A and core region are consistent with LPS found in *E. coli* K-12; however, this strain does not naturally produce an O-antigen, whereas many environmental and clinical strains do. Strains lacking the O-antigen region are said to contain “rough” LPS, and this can further be divided into subtypes dependent on truncations in the core region. The most extreme of these that is still viable at 37 °C under laboratory growth conditions is “deep rough” LPS, containing only lipid A-Kdo_2_. The O-antigen region is highly variable within species and can contain as many as 40 glycan repeats. *Kdo*, keto-deoxyoctulosonate; *LDmanHep*, l-glycero-d-manno-heptose; *Glc*, glucose; *Gal*, galactose; *P*, phosphate group; *PEtN*, phosphorylethanolamine.

The OM of Gram-negative bacteria is unusual in that it is a highly asymmetric lipid bilayer, comprising an inner leaflet enriched in phospholipids and an outer leaflet containing lipopolysaccharide (LPS) (Fig. 2) (99). This is in contrast to the IM in Gram-negative and Gram-positive bacteria, which mostly contain phospholipids mixed between both leaflets. In Gram-negative bacteria, phospholipids in the OM generally have the canonical structure expected of a phospholipid, containing two hydrophobic acyl chains, with different length and degree of saturation. These are connected via an ester linkage to a headgroup that can be zwitterionic, or positively or negatively charged (Fig. 2). Cardiolipin (CL) is also found in the OM and has the appearance of a phospholipid dimer (Fig. 2). LPS is a much bulkier molecule, made up of a variable number of acyl chains (between 4 and 8, depending on the species) (100). The acyl chain can vary in length both within each molecule and between species (*e.g.* C_10:0_–C_14:0_ in *Bordetella pertussis*, C_12:0_–C_14:0_ in *E. coli*, C_14:0_–C_21:0_ in *Chlamydia trachomatis*, and C_14:0_–C_28:0_ in *Agrobacterium tumefaciens*) (101–104). Furthermore, the acyl chains of LPS are usually shorter than those of the average phospholipid and are almost always saturated (105) (Fig. 2). The acyl chains in LPS are connected to a disaccharide diphosphate headgroup, which in turn is connected to a conserved “core” region made up of chained sugar groups, and then finally a highly variable sugar-containing O-antigen region (Fig. 2) (106, 107). Together, these sugar regions convey a large net negative charge to the outer surface of bacteria (107).

### Essentiality of specific lipids

*E. coli* is remarkably tolerant of modifications in its lipid biosynthesis pathways, with viable strains including bacteria in which synthesis of phosphatidylethanolamine (PE) (108, 109), phosphatidylglycerol (PG) and CL (110, 111), or CL alone (109, 112) is eliminated (see Fig. 2 for the structure of common lipid types); phosphatidylcholine (PC) synthesis is induced synthetically (*E. coli* lacks PC in its IM or OM, although this phospholipid is present in some bacterial membranes) (113, 114); gluco- or galactolipids are utilized (115); or even archaeal lipids are incorporated into the membrane (116). Although these strains are able to survive under laboratory conditions, their growth and virulence are affected (in some cases severely), stress responses are up-regulated, and defects of varying acuteness are seen in the structure and permeability of the cell envelope (109, 117). The effect of such changes in lipid composition in the OM on OMP biogenesis has not been investigated in detail for all of these strains. However, in PE-deficient strains, OmpF folding is impaired in a titratable manner, with complete lack of PE reducing folding yields from ∼100% in WT to <15% (109). Lack of CL causes less severe defects but still reduces OmpF folding yields to ∼25% (109) and has also been shown to cause mislocalization of the OMP IcsA, which normally resides at the cell pole in *Shigella flexneri* (117). Interestingly, in *E. coli*, lack of CL causes severe distention/detachment of the OM from the IM at the cell poles, and CL and PG have been observed to accumulate at cell poles and division sites (118), suggesting a role for CL in maintaining cell shape and integrity at sites of negative curvature (119). PG null only mutants have not been described, as CL utilizes PG for its biosynthesis. However, the creation of viable strains absent in PG synthesis also requires mutations in the major *E. coli* OM lipoprotein Lpp (Braun's lipoprotein), suggesting that lack of PG causes lethality primarily though lethal accumulation of Lpp at the IM (111, 120). The first step in lipoprotein maturation after translocation into the periplasm involves the transfer of a diacyl moiety from PG (121), and its absence presumably stalls maturation at this point. However, the OM lipoproteins LptE and BamD are essential in *E. coli*, so the viability of these *lpp* mutant strains suggests that alternate maturation pathways or sources of diacylglycerol must exist (122, 123). As BamA and LptD are essential OMPs, the fact that bacteria can still grow and divide in these strains (albeit poorly) suggests that other lipids can moonlight for the loss of PE, PG, or CL or that there is no absolute need for a particular phospholipid type as a minimum requirement for OMP biogenesis in *E. coli*. Nonetheless, the severe defects observed in these strains show that outside the laboratory, all of these components are needed for bacterial viability. This highlights that whereas a stable bilayer is the minimum requirement to fold an OMP, to understand how OMP biogenesis occurs in biologically relevant environments, consideration of the complexity of the OM environment is crucial.

### Organization of lipid types within the OM

**Figure 3.**
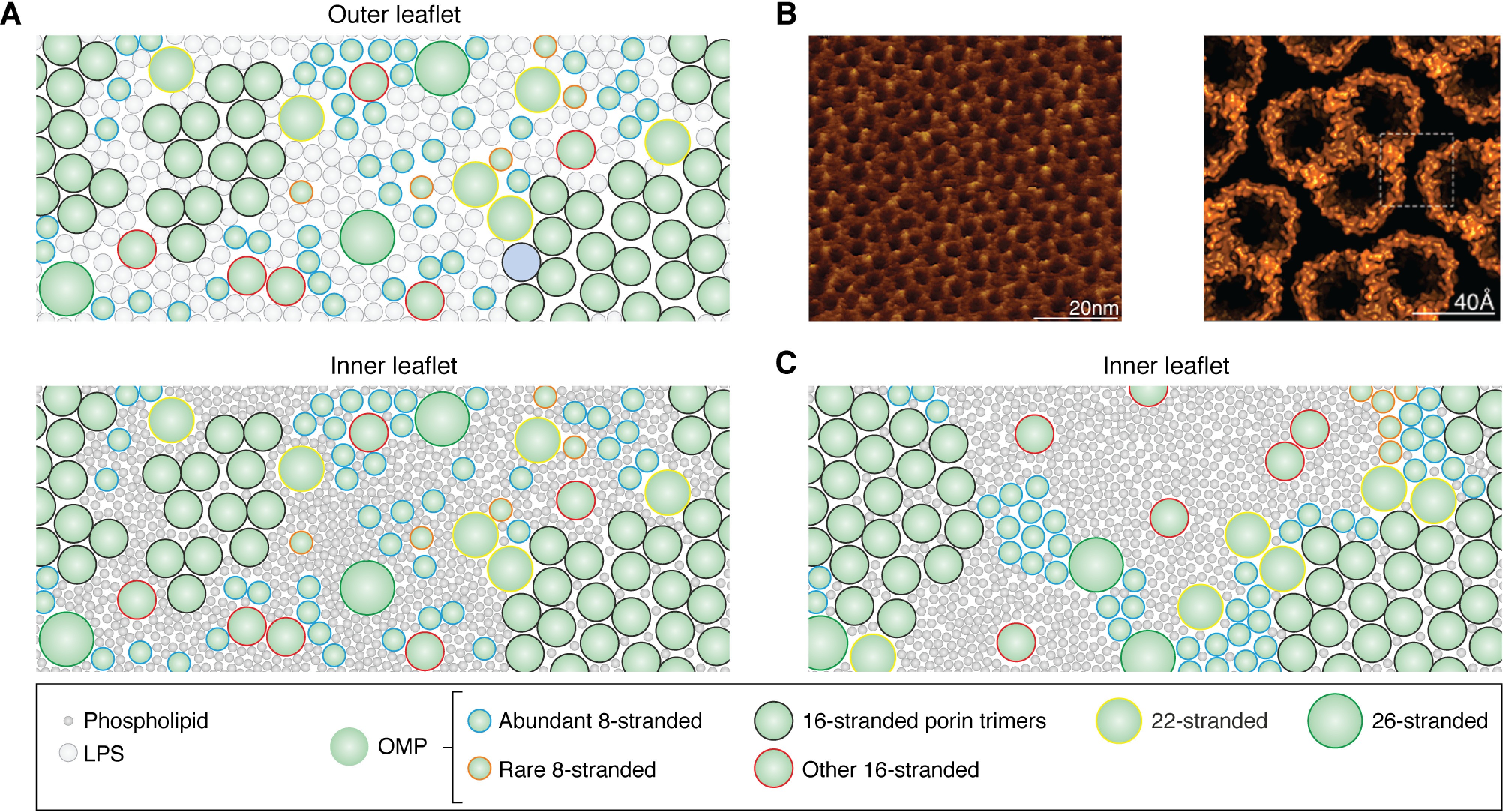
**Model depicting the structural organization of the *E. coli* OM.** A schematic displays the degree of crowding in the OM. *A*, view of an imagined OM showing the dense packing of different size OMPs in monomers, dimers, and trimers interspersed with LPS in the outer leaflet (*top*) and phospholipids in the inner leaflet (*bottom*). Phospholipids are represented as *dark gray circles* with a diameter proportional to the headgroup size of PE/PG and LPS as *light gray circles* with a diameter proportional to the size of lipid A. Different OMPs are represented as *idealized circles* with diameters proportional to their number of strands. *Blue outline*, abundant 8-stranded; *orange outline*, rare 8-stranded; *black*, 16-stranded porin trimers; *red*, other 16-stranded; *yellow*, 22-stranded; *green*, 26-stranded. The overall LPR in this schematic is ∼9:1, with ∼2 LPS and ∼7 phospholipids per OMP, consistent with estimates for the LPR of the *E. coli* OM. *B*, *left*, high-resolution AFM image of OM extracts from *Roseobacter dentrificans* imaged from the periplasmic side showing a dense lattice of porin trimers. *Right*, atomic model of the packing of porin trimers derived from the AFM data. Reproduced with permission from Jarosławski *et al.* (156). This research was originally published in Molecular Microbiology. Jarosławski, S., Duquesne, K., Sturgis, J. N., and Scheuring, S. High-resolution architecture of the outer membrane of the Gram-negative bacteria *Roseobacter denitrificans*. *Molecular Microbiology* 2009; 74:1211–1222. © Wiley–Blackwell. *C*, view of an imagined OM with the same LPR as in *A* but assuming a more extreme clustering of most OMPs. Only the inner leaflet is shown. Despite having the same LPR values, the buried surface area of the clustered OMPs frees up more lipid to form larger bulk lipid domains.

The *E. coli* OM contains PE, PG, CL, and LPS (Fig. 2). These lipid types are divided asymmetrically between the inner and outer leaflets of the OM, with the outer leaflet containing almost 100% LPS and the inner leaflet containing ∼80% PE, ∼15% PG, and ∼5% CL (Figs. 2 and 3). By contrast, the IM also contains ∼5% CL with a lower ratio of PE/PG of ∼70%/25% (124). Although it is physically possible for phospholipids to flip from the inner leaflet to the outer leaflet, this process is likely to be intrinsically slow (occurring on the order of hours or longer in vesicles *in vitro*) (125, 126). However, this process may be accelerated under conditions of OM/bilayer stress, such as exposure to antimicrobial peptides or detergents, in strains with truncated LPS (see Fig. 2), or after loss of OMPs (78, 127–129). This process is therefore associated with increased permeability of the OM. Regardless of such events, the asymmetry of the OM is actively maintained in *E. coli* by the maintenance of lipid asymmetry (Mla) system, which removes errant phospholipids specifically from the outer leaflet of the OM to maintain its barrier function (130–132).

### The lipid acyl chain composition is diverse

The acyl chain composition of lipids in the OM of *E. coli* is more variable than that of their headgroups. The acyl chain composition of the OM depends on the growth conditions, with acyl chains varying in length from C_12_ to C_18_, as well as in the degree of saturation or the presence of cyclopropyl modifications (Fig. 2) (133–138). Lipidomics has provided insights into the range of acyl groups found in *E. coli* membranes. Early experiments in *E. coli* K1062 reported the presence of C_12:0_, C_14:0_, C_16:0_, C_16:1_, C_18:1_, and cyclo-C_19:0_ acyl chains in phospholipids from the IM and OM (139). Examining total lipid content in *E. coli* K-12 strain LM3118 grown at 37 °C and harvested in stationary phase showed that the acyl chains of PE and PG lipids were comprised primarily of C_12:0_, C_14:0_, C_16:0_, C_16:1_, C_18:0_, and C_18:1_ (with C_16:0_ being about 3 times more abundant than the other acyl chains), with a lesser contribution from C_15:0_, cyclo-C_17:0_, and cyclo-C_19:0_ (140, 141). These acyl chains are combined to form a large variety of phospholipid types, most containing at least one unsaturated acyl bond or cyclo-propyl group, although *di*C_16:0_PE, *di*C_16:0_PG, C_16:0_C_14:0_PG, C_16:0_C_12:0_PE, *di*C_14:0_PE, and *di*C_12:0_PE lipids were also observed. Despite cyclo-propyl acyl chain–containing lipids being relatively understudied, the most common lipid species detected under these conditions was C_16:0_/cyclo-C_17:0_ (Fig. 2). Cyclo-propyl–containing lipids are produced in large quantities in stationary phase cultures from the conversion of double bonds in unsaturated chains to cyclo-propyl groups (142, 143). Although the physiological functions of these modifications are unclear, they appear to be related to protection of the bacteria against a variety of adverse environmental conditions (143), including acid shock (144, 145), osmotic shock (146), high alcohol concentrations (147), or high temperature (148). Furthermore, OMP folding in *E. coli* occurs primarily during exponential growth (56, 149), while expression is down-regulated (150, 151) and OMPs are lost from the OM (152) when bacteria enter stationary phase. Thus, the relevance of this lipid modification for OMP biogenesis may be minor under favorable growth conditions.

Few studies have examined whether significant differences or biases exist between the acyl chain composition of phospholipids in the IM and those of the OM of Gram-negative bacteria. Some have reported an enrichment of shorter acyl chains (C_12:0_ and C_14:0_) (139), saturated fatty acids (153), lyso-PE lipids (154), and C_16:0_ acyl chains in the OM and a depletion of polyunsaturated acyl chains (155). These biases, however, generally vary with growth conditions, and it is unclear to what extent this simply reflects the presence of the lipid A component of the outer leaflet. Regulatory systems that alter the acyl chain composition of lipids specific to the OM are known for LPS, including enzymes that alter the acyl chains attached to lipid A to modulate the endotoxicity of this lipid type during growth in a host (99). Modulation of the acyl chain content of lipid A has also been shown to occur in *E. coli* when under selective pressure from an external insult by the addition of a bactericidal BamA-specific antibody. This suggests a direct link between modulation of lipid content and a selective pressure to efficiently fold OMPs (93). However, it is not clear whether this change in lipid A reflects a need to aid the function of an essential BAM client (*e.g.* LptD), is related to conformational changes in BamA, or is simply a response to a defective permeability barrier.

This diversity of acyl chain types gives *E. coli* a wide range of lipids with which it can tailor the biophysical properties of its membranes both globally and locally. This variety may allow it to deal with local minor deformations of the membrane, due to either random thermal fluctuations or the presence of membrane-bound or embedded proteins, as acyl chains can diffuse laterally and occupy the most energetically favorable position, dependent on the match between their own physicochemical properties (length and saturation) and those of the membrane environment.

### A crowded environment

**Figure 4.**
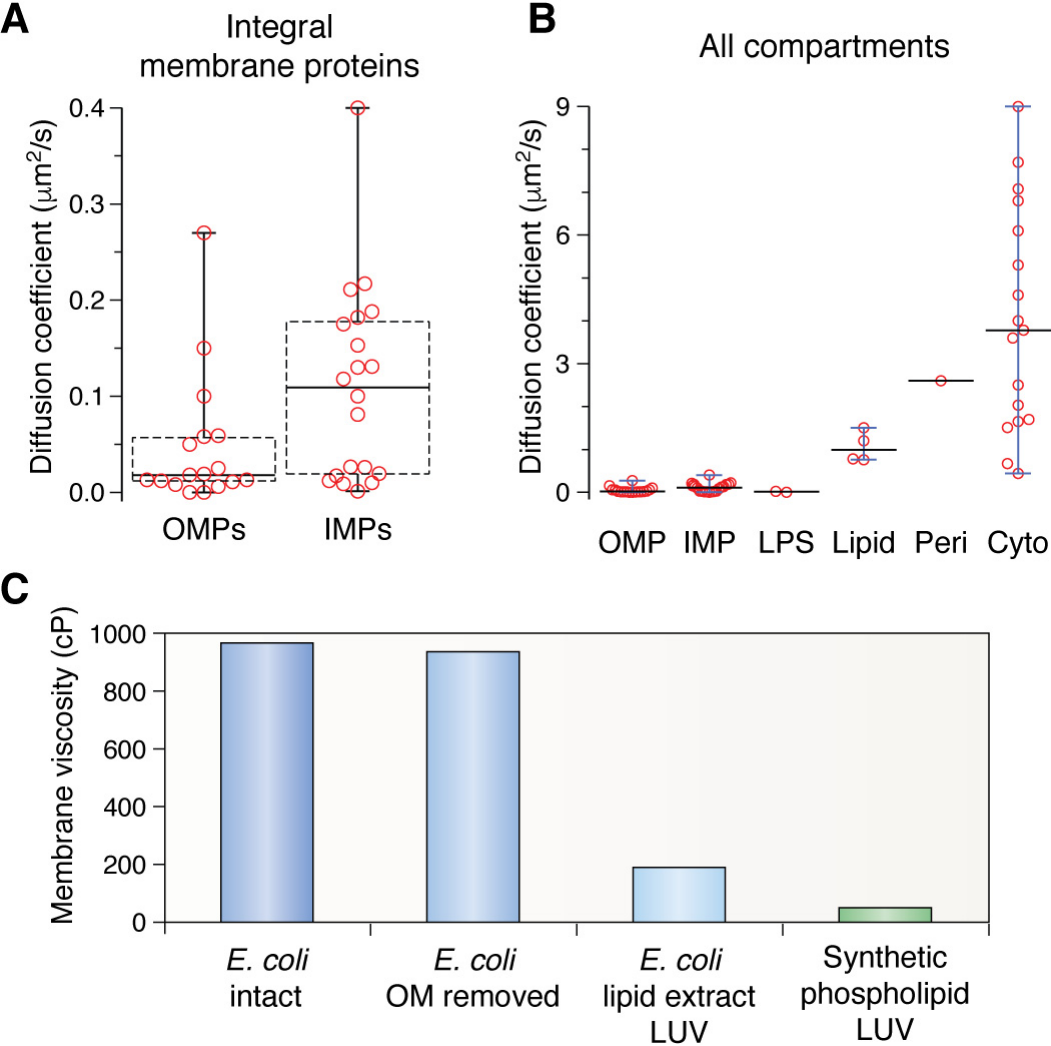
**Comparison of physical properties of bacterial membranes.** *A*, box plots showing the range of diffusion coefficients reported for OMPs and IMPs (see “*A crowded environment*”). Boxes show interquartile range calculated by the Tukey method with the median indicated as a *boldface horizontal line*. *Whiskers* show the minimum and maximum values. *B*, comparison of the diffusion coefficients of membrane proteins with other components of bacteria. *Whiskers* are only shown for components that have three or more values reported in the literature. All values are reported from *in vivo* studies. *LPS*, diffusion of LPS molecules in *S. typhimurium*. *Lipid*, diffusion rate of a fluorescent lipid reporter probe in *E. coli* membranes. *Peri*, diffusion of soluble protein in the *E. coli* periplasm. *Cyto*, diffusion of soluble protein in the *E. coli* cytoplasm. *C*, viscosities of different membrane environments as measured by the use of fluorescent BODIPY C10 lipid reporter probes. *E. coli* data are from Mika *et al*. (175), and synthetic phospholipid data are from Wu *et al*. (225). BODIPY C10 specifically incorporates into the IM of *E. coli*, and removal of the OM minimally affects the measured viscosity. Synthetic phospholipid 200 nm LUVs were comprised of DLPC, DMPC, POPC, or DOPC.

Although the familiar fluid-mosaic model of membranes found in most textbooks depicts a biological membrane with just a few proteins floating in a “sea” of lipid, the OM is markedly different, containing instead a much higher fraction of protein by weight, with lipid/protein ratios (LPRs) (w/w) estimated to be between 0.14 and 0.36 (139, 156), corresponding to only 2–4 LPS and 4–10 phospholipid molecules per OMP (157). Estimates based on biochemical studies suggest that as much as 50% of the surface area of the OM may be occupied by OMPs (7), whereas AFM studies (156) (Fig. 3*B*), extrapolation from the copy numbers of OMPs measured by proteomics (3, 4), and the above measurements of the LPR suggest that this value may be even higher. For example, a copy number of 100,000 for OmpA would imply that ∼6–20% of the surface area of *E. coli* (dependent on the size of the bacterium) would be occupied by this protein alone. Hence, the OM could be considered more like a protein-rich layer solubilized in a relatively small amount of lipid (Fig. 3, *A* and *B*). Despite the low LPR of the OM, the diffusion rates of OMPs in the OM of *E. coli* are similar to those of inner membrane proteins (IMPs) but are, on average, slower (diffusion coefficients of 0.006–0.15 μm^2^/s for OMPs *versus* 0.001–0.4 μm^2^/s for IMPs) (56, 158–170) (Fig. 4*A*). For comparison, the length elongation rate of *E. coli* alone is ∼0.006 μm/s (171), whereas the diffusion coefficients of LPS in the OM of *Salmonella typhimurium* are ∼0.00005 and 0.02 μm^2^ s^−1^ (for O-antigen–containing and truncated “deep rough” LPS, respectively (Fig. 2)) (172, 173), lipid probes in the IM of *E. coli* ∼0.8-1.5 μm^2^/s (162, 174, 175), and the periplasm, cytoplasm, and buffer ∼3, 0.4-9, and ∼87 μm^2^/s, respectively (159, 168, 176–180) (Fig. 4*B*). What particularly distinguishes OMPs from IMPs is their restricted diffusion areas, with diffusion being confined within clusters in the OM, compared with free diffusion of most IMPs in the IM (56, 166, 181, 182). These observations can be explained by the propensities of OMPs to form clusters (56, 149, 156, 181–188) and/or to interact strongly with LPS or components of the cell envelope (189, 190). OMPs that have a lower tendency to cluster and/or interact with cell envelope components less strongly may exhibit higher diffusion coefficients but will ultimately become “corralled” within OMP-LPS domains. On this point, an abundance of clustered OMPs with low mobility may make the OM locally rigid. Indeed, molecular dynamics (MD) simulations have shown that membranes containing 8–12-stranded OMPs are much stiffer than membranes containing only DMPC (*di*C_14:0_PC) (157). However, simulations investigating larger length scales have shown that crowding a bilayer with some OMPs (*i.e.* BtuB), but not others (*i.e.* OmpF), can reduce the global bending rigidity of a POPE (C_16:0_C_18:1_PE)/POPC (C_16:0_C_18:1_PC) membrane (191)—an effect that would explain how a cell with a protein-rich OM could still maintain its shape. An interesting alternative possibility to explain the potentially incompatible concepts of OMP folding and a protein-dense, lipid-poor OM would be to consider the OM as an inhomogeneous mixture of protein and lipid (Fig. 3*C*). The view of OMP monomers or trimers, well-solubilized with lipid, would leave little bulk lipid available for nascent OMPs to fold. However, by clustering OMPs together into regions resembling two-dimensional crystals (*i.e.* forming regions highly enriched with OMPs and little to no lipid—a local LPR closer to 1:1 (mol/mol)), sufficient lipid-rich regions would be created to enable OMP folding and insertion (Fig. 3, *B* and *C*). Regardless of which model is correct, this locally stiff, crowded, and confined environment, with a relative paucity of free lipid, poses a challenging environment into which OMPs must fold.

## More than a mix of lipid types

### The physical properties of lipid membranes

**Figure 5.**
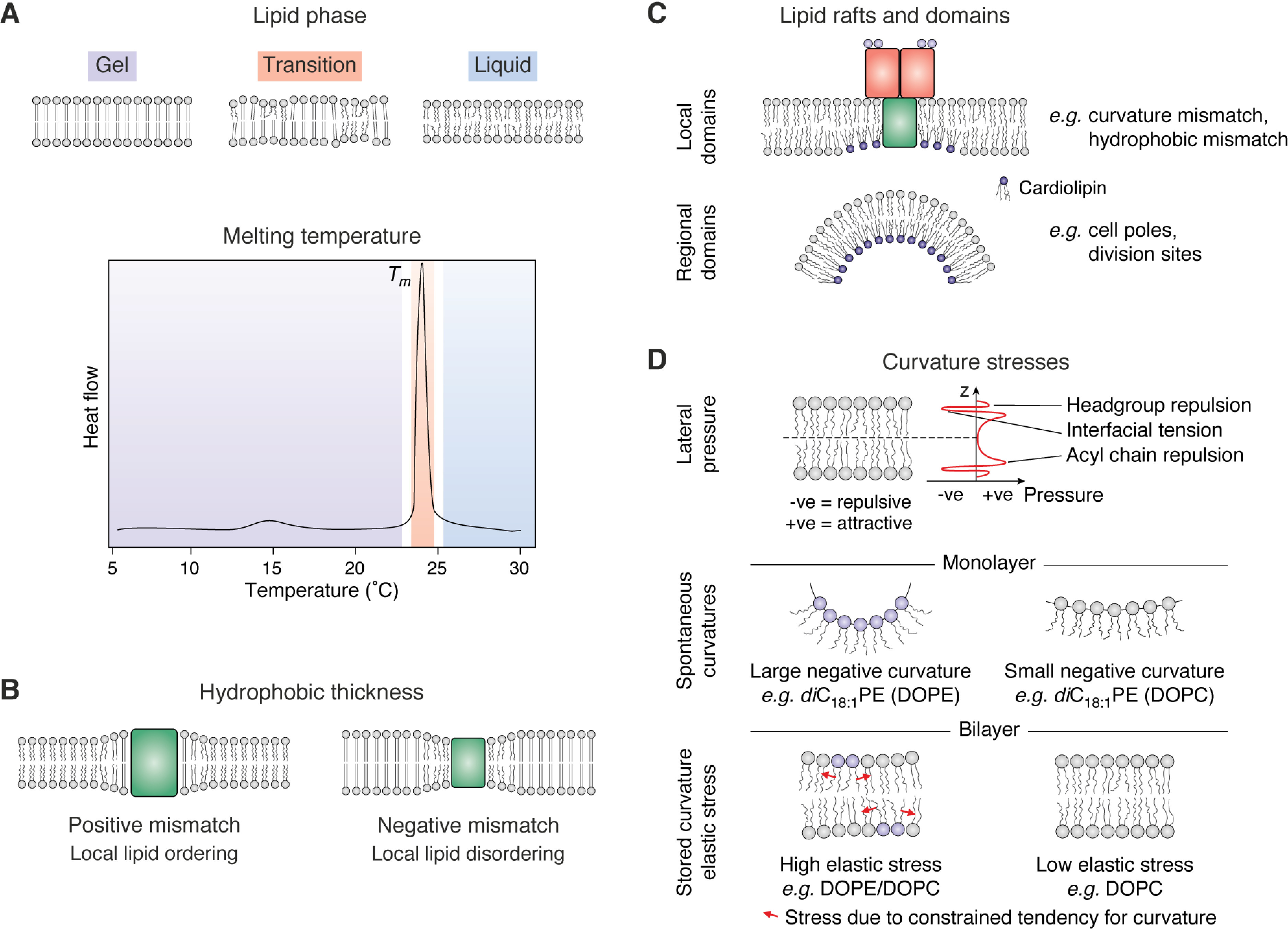
**Physical and mechanical properties of a lipid bilayer.** *A*, *top*, the phase of a lipid bilayer depends on the temperature, with the lipids being in an ordered (gel) phase below the *T_m_* and in a (liquid) disordered phase above the *T_m_*. At the transition temperature, frustration between packing of regions of gel and liquid phase causes defects to occur at these boundaries. *Bottom*, a typical differential scanning calorimetry curve illustrating the thermal response of a DMPC (*di*C_14:0_PC) bilayer with the regions of each phase *colored* as above. *B*, the hydrophobic thickness of a membrane depends on the lipid acyl chain length. However, when an OMP becomes embedded in a lipid bilayer, the membrane responds by trying to “match” the hydrophobic thickness of the bilayer to that of the protein to minimize the energetic penalty of exposing polar lipid headgroups to a hydrophobic OMP surface or hydrophobic acyl tails to polar OMP loops. *C*, mixtures of lipids can separate, forming “rafts” or domains dependent on the physical conditions and lipid type. CL has a high propensity for negative curvature and has been shown to be enriched at cell poles and division sites where the membrane constricts. CL has also been observed to bind to membrane protein complexes such as the BAM complex (397) and cluster under patches of LPS in MD simulations (209, 210), suggesting that it may help stabilize bilayer packing defects (which might be induced by LPS) and stabilize regions of large hydrophobic mismatch (*e.g.* around embedded proteins such as BamA). *D*, schematic describing stored curvature elastic stress and how this depends on lipid type. Adapted from Booth and Curnow (398). This research was originally published in Current Opinion in Structural Biology. Booth, P. J., and Curnow, P. Folding scene investigation: membrane proteins. *Current Opinion in Structural Biology* 2009; 19:8–13. © Elsevier. Attractive and repulsive interactions driven by the packing of lipids create a pressure differential along the normal of the membrane that must be overcome to deform or alter lipid packing. Incorporation of lipids that have a tendency toward negative curvature (due to the relative size of the headgroup *versus* the acyl chain (*e.g.* PE lipids)) into a bilayer formed from lipids with a neutral or low curvature tendency (*e.g.* PC lipids) generates a stress force within the bilayer due to the opposing tendencies for bilayer formation of these lipids.

Lipid bilayers can be characterized by a number of physical, mechanical, and chemical parameters, including stored curvature elastic stress (lateral pressure), melting temperature, the bulk lipid phase, the presence of lipid rafts, membrane viscosity, and headgroup charge (192–194) (Fig. 5). Many of these properties are interrelated and can be modulated by altering the acyl chain composition and/or the phospholipid headgroup and by altering the relative amounts of phospholipid, CL, and LPS (195–199). Stored curvature elastic stress makes membranes more rigid and less elastic in terms of their ability to deform or bend. This property can be introduced by the presence of nonbilayer-forming lipids in otherwise bilayer-forming membranes (Fig. 5). For example, PE lipids create negative curvature, whereas PG and PC lipids have zero or low spontaneous curvature, which allows them to readily form bilayers (200). Doping bilayers containing PC or PG with PE or CL generates a tension in packing of the different lipid types, creating a crowding, or pressure, near the center of the bilayer where each leaflet meets (Fig. 5), which is further altered by the length of the acyl chains (with shorter chains reducing this elastic stress) (200).

The lipid phase of a membrane is also dependent on its lipid composition and on the temperature (Fig. 5). Bilayers exist primarily in one of two major states, a solid “gel” phase in which the acyl chains are tightly packed and the mobility of lipid molecules is low, and a “liquid” phase, where lipid mobility is higher (201–203). Furthermore, analogous to the familiar phase change of ice to water, lipids in a gel-phase membrane can “melt” to become the liquid phase at a temperature characteristic of the particular lipid type—called the transition temperature, *T_m_*. Lipid mixtures can also adopt a “liquid-ordered” phase (with the classical pure liquid phase described as “liquid-disordered”). This liquid-ordered phase contains lipids that are highly mobile but have well-ordered acyl chains and is often associated with the formation of lipid rafts in cholesterol-containing membranes in eukaryotes (204–206). Although sterol lipids are rare in bacteria, CL may play a similar role in increasing lipid order, and there is evidence that CL can participate in the formation of rafts/domains in membranes *in vitro* and *in vivo* (Fig. 5) (although it is likely this mechanism is distinct from that of cholesterol) (207, 208). *In silico*, CL has been observed to form clusters under patches of LPS in simulations of bacterial OMs in a role that may compensate for packing defects in the outer leaflet of the OM (209, 210).

### The physical properties of native lipid extracts

The OM differs from the IM in its highly asymmetric structure, large fraction of proteins by weight, permeability to small molecules (<600 Da), and lack of energization across it (11, 99, 139, 156, 211). However, relatively little is known about how these unique features of the OM affect its mechanical properties and how this differs from the IM. *E. coli* is known to alter the lipid content of its membranes, particularly the length and degree of saturation of acyl chains, in response to changes in growth temperature. This process, termed “homeoviscous adaptation” (212), suggests that bacteria actively maintain their membranes at a constant level of “fluidity,” or in a particular phase, that enables them to adjust to different environments. For example, whereas total lipid extract from *E. coli* K-12 W3110 showed approximately the same headgroup content (with a minor monotonic increase in CL and decrease in PG) when grown at 30, 37, 42, or 45 °C (213), the ratio of saturated over unsaturated acyl chains increased with temperature. This suggests a change to a more “rigid” mixture of phospholipids at higher growth temperatures to balance the increased fluidity caused by the input of thermal energy.

Membrane lipid properties can be probed using fluorescent reporter dyes that partition into membranes (either globally or into specific lipid phases) and alter their excitation or emission properties according to the local lipid environment. Hence, these dyes can be used as reporters of membrane viscosity, degree of hydration, phase, and mobility (214). The fluorophore laurdan partitions into membranes through its acyl tail, whereas its naphthalene-based headgroup resides in the interfacial region of bilayers, where its fluorescence excitation and emission spectra are sensitive to the degree of hydration of the bilayer, allowing it to report on the phase and order of lipids in a bilayer (215). Using laurdan, a *T_m_* of *E. coli* total lipid extract of <14 °C was determined for bacteria grown at 30 °C or 37 °C, whereas the *T_m_* was higher (*T_m_* ∼20-22 °C) for bacteria grown at 42 °C and elevated again (*T_m_* ∼27 °C) for bacteria grown at 45 °C (213). This shows that the lipids in *E. coli* are natively in the liquid phase, and their *T_m_* is as much as 20 °C below the growth temperature. However, when the OM and IM are considered separately, it becomes clear that “global” lipid properties do not accurately capture the differences between these two different membranes. Deuterium NMR studies, used to measure the order of acyl chains, found that lipids in an OM preparation of *E. coli* L51 were less fluid than those from the IM and that the *T_m_* was ∼7 °C higher than the IM (216, 217). Electron spin resonance experiments on IMs and OMs of *E. coli* W1485 doped with a spin-labeled stearic acid probe found the *T_m_* of the OM (26 °C) to be ∼13 °C higher than the IM (13 °C) when the bacteria were grown at 37 °C (196). Fluorescence polarization studies using IM and OM extracts from *E. coli* B doped with parinaric acid also found that the phase transition of the OM was ∼15 °C higher than the IM, initiating its phase transition at 40 °C (218).

### E. coli membranes in situ

Few studies on the properties of bacterial membrane lipid order (or phase) *in vivo* have been reported to date, but current data suggest that the organization of the OM is more complicated than that derived using lipid extracts, as described above. Differential scanning calorimetry studies on whole cells of *E. coli* W945 grown at 20 or 37 °C observed two reversible transitions, one well below the growth temperature, which was suggested to correspond to the IM, and another slightly above the growth temperature, which was assigned to the OM (219). Experiments on fixed cells using laurdan and the dye 1,3-diphenyl-1,3,5-hexatriene as a probe of local viscosity (214) have shown that *E. coli* membranes are predominantly in the liquid phase. These experiments also revealed that these membranes are heterogeneous and contain at least two distinct phases, one more liquid and one less so, possibly indicating the presence of distinct lipid domains (220). However, an alternative explanation of these data is that they reflect a difference between the IM and the OM, because it is not clear to which membrane these probes localize. Indeed, whereas early studies suggested that these dyes (and others, including FM 4-64) localize primarily to the IM (221), it is now known that FM 4-64 partitions specifically into the OM immediately after labeling (19), and 1,3-diphenyl-1,3,5-hexatriene may equilibrate between both membranes (222, 223) or be trapped mainly in the first hydrophobic surface encountered (224). Measurements of membrane viscosity using “molecular rotor” dyes such as BODIPY C10 (viscosity alters the fluorescence lifetime of the probe) showed that the IM of *E. coli* is more viscous than previously believed, with an average viscosity of 980 cP for intact *E. coli*, 950 cP for spheroplasted cells at 37 °C, and 200 cP for large unilamellar vesicles (LUVs) of *E. coli* total lipid extract at 37 °C (175). By contrast, a lower viscosity (∼60 cP) was consistently observed for 200-nm LUVs formed from DLPC (*di*C_12:0_PC), DMPC (*di*C_14:0_PC), POPC (C_16:0_C_18:1_PC), or DOPC (*di*C_18:1_PC) in their liquid phase at 37 °C (225) (Fig. 4*C*). Although not yet measured directly, the lower diffusion coefficients of OMPs compared with IMPs (Fig. 4, *A* and *B*) suggest that the viscosity of the OM may be even higher still, and this is a characteristic that model lipid membranes commonly used for OMP-folding studies (see below) clearly cannot capture.

### The influence of LPS on the mechanical properties of the OM

On the basis of the physicochemical properties of LPS extracts (which generally show *T_m_* values at or above the growth temperature) (226–230) and those of the OM (discussed above), some authors have argued that the OM is more likely to exist in the gel phase at physiological temperatures (78). However, the exact thermotropic response of LPS in the outer leaflet may vary, depending on the composition (particularly the presence of hydrogen bond donors and acceptors) and size of the polysaccharide region of the LPS molecules incorporated (78, 229, 231, 232). *In vitro* models of the OM using an asymmetric bilayer deposited on silicon or lipid-coated gold surfaces showed that the membrane has unusual mixed characteristics with elements of both liquid- and gel-phase lipids (233, 234). The OM in these studies comprised “rough” LPS (containing the conserved polysaccharide core but lacking the O-antigen) (Fig. 2) in the outer leaflet and phospholipid (DPPC (*di*C_16:0_PC)) in the inner leaflet, with lipid order parameters measured using neutron reflectometry and attenuated total reflection FTIR spectroscopy (ATR-FTIR) (233). Two transition midpoints (*T_m_*) were observed, one just below (∼36 °C) the physiological growth temperature of *E. coli* (37 °C) for the outer leaflet of LPS and the other above the *T_m_* (∼39 °C) for the inner leaflet comprising DPPC (*di*C_16:0_PC) (233). Even though the composition of the inner leaflet lipids and LPS differ *in vivo* from those used in this study, the results suggest that the LPS component of the asymmetric OM may confer greater rigidity to the OM. This agrees with deuterium NMR studies of preparations of *E. coli* OM and IM, which found that at a given temperature, phospholipids were more ordered and a larger fraction were in the gel phase in the OM than the IM (217, 235). However, it should be noted that extraction of the OM likely causes mixing of the inner and outer leaflets, reducing the asymmetry (236).

The fluidity of the OM of *E. coli* may also be controlled by temperature-dependent modification of LPS in the outer leaflet. The *lpxT* gene in *E. coli* transfers a phosphate group onto lipid A, which may alter the rigidity of the OM, and expression of this gene is regulated by an mRNA thermostat (237). LpxT is an IM protein that covalently modifies LPS before it reaches the OM. The protein was shown to be stable between 28 and 42 °C; however, its mRNA levels fall dramatically across this same temperature range (237). Another LPS biosynthesis pathway gene, *lpxP*, replaces the C_12:0_ chain in *E. coli* lipid A (which is normally installed by *lpxL*) with a C_16:1_ chain, but expression of this protein is only induced at low temperature (12 °C) and may also be regulated by an mRNA thermostat similar to *lpxT* (238). *Francisella novicida* synthesizes lipid A with shorter acyl chains at low growth temperatures (25 °C compared with 37 °C), an effect that has been linked to both the differential expression and enzyme activity of the *lpxD2* and *lpxD1* genes (which add different length acyl chains to lipid A) at 25 and 37 °C, respectively (239). In *Yersinia pestis*, the *lpxR* gene, which is responsible for the removal of acyl chains from lipid A, is inactive at 21 °C (resulting in a hexa-acylated LPS) but functional at 37 °C, resulting in a tetra-acylated LPS in the OM of bacteria grown at this temperature (240). The deacylase enzyme and OMP, PagL, alters lipid A through the removal of acyl chains. The activity of this enzyme in *Pseudomonas aeruginosa* is also affected by growth temperature, with activity falling at low growth temperature (≤ 21 °C) (241). The extent to which these temperature-dependent modifications are a mechanical response to maintain or alter membrane rigidity/fluidity or an immune-modulating response triggered by the detection of a host environment, or both, remains unclear (242). Despite this, it is likely that the structure and mechanical properties of LPS are important for OMP folding. For example, genetic alterations to the biosynthetic pathway of LPS, which cause changes in its degree of acylation or its sugar headgroup have been shown to cause severe defects in the biogenesis of OMPs (243–245).

### The effect of asymmetry

The asymmetric architecture of the OM, with an inner leaflet containing canonical phospholipids and an outer leaflet containing LPS with its highly acylated lipid A attached to large sugar groups that protrude into solution, impacts the physical properties of the OM and creates a lipid environment that is very different from that of the IM *in vivo* and vesicles of lipids commonly used in *in vitro* experiments. Representative models of the OM have been built *in silico* and studied using coarse-grained molecular dynamics (CG-MD) and atomistic molecular dynamics (A-MD). A model was built of the *P. aeruginosa* PAO1 OM, with an LPS outer leaflet and a DPPE (*di*C_16:0_PE) inner leaflet and studied by A-MD (246). At 37 °C, molecules of DPPE in the inner leaflet showed diffusive movement consistent with a liquid phase, whereas LPS in the outer leaflet showed an order of magnitude lower mean-square displacement. Despite this low lateral mobility, calculation of the lipid order parameters of the acyl tails of LPS indicated that they are fluid and not ordered as would be expected for lipids in the gel phase (246). Similar results have been observed in other A-MD simulations of *E. coli* and *P. aeruginosa* OMs with an outer leaflet containing LPS with a short O-antigen region (247, 248) or a “rough” LPS (248, 249) (Fig. 2) and an inner leaflet comprised of a 75:20:5 (mol/mol/mol) mix of PPPE (C_16:0_C_16:1_PE)/PVPG (C_16:0_C_18:1_PG)/CL. CG-MD simulations of the *E. coli* OM with a DPPE (*di*C_16:0_PE) inner leaflet and an outer leaflet containing a different ratio of LPS/DPPE (*di*C_16:0_PE) from 10:90 to 100:0 (mol/mol) showed that increasing the fraction of LPS lowered the simulated *T_m_* from 73 to 15 °C (250) (for reference, a symmetric bilayer composed solely of DPPE has an experimental *T_m_* of ∼64 °C, showing interleaflet coupling can have hard-to-predict consequences on phase behavior) (251). A-MD simulations of a model of a *P. aeruginosa* OM containing only lipid A in the outer leaflet and a mix of DPPE (*di*C_16:0_PE), DPPG (*di*C_16:0_PG), DOPE (*di*C_18:1_PE), and DOPG (*di*C_18:1_PG) in the inner leaflet showed that the acyl chains of lipid A were also consistent with a liquid phase but were overall less disordered than observed with larger sugar regions—again emphasizing the importance of the polysaccharide region of LPS in modulating its packing behavior (252). Other CG-MD experiments showed that *E. coli* asymmetric membranes with a lipid A outer leaflet and a DPPE (*di*C_16:0_PE) inner leaflet had a lower *T_m_* than a “rough” LPS outer leaflet and DPPE (*di*C_16:0_PE) inner leaflet (∼41-46 °C *versus* ∼55 °C) (253), although it is not clear why the *T_m_* values are much higher in these simulations than observed previously from the same group (250). Preparations of the OM and IM from *E. coli* J5 doped with spin label probes showed that membrane order is higher, and lipid mobility lower, in the OM, and the magnitude of this difference decreases when a large fraction of LPS is removed (254). Furthermore, in the absence of galactose, this strain is unable to synthesize the full polysaccharide region and produces a short “rough” LPS (Fig. 2) (255). The presence of the O-antigen was shown to confer even greater rigidity to the OM extracts than when they contained only truncated “rough” LPS (254). Deuterium solid-state NMR experiments on multilamellar vesicles containing a mix of partially deuterated DPPC (*di*C_16:0_PC) and “rough” LPS from *E. coli* J5 or *E. coli* EH100 also found an “ordering” effect on the acyl chains of DPPC conferred by LPS (256). Despite this ordering of acyl chains, experiments with SUVs composed of *E. coli* B LPS and phospholipid extract doped with spin-labeled PE and PG lipids, and *S. typhimurium* OM lipid preparations doped with a spin-labeled stearic acid probe found that phospholipids remained freely diffusive and segregated away from LPS (226, 257, 258). The slower diffusion of phospholipids in the inner leaflet of the OM observed in the previous study (which retained its complement of OMPs) (254) may therefore be due to the transient clustering and reduced diffusion of lipids around embedded OMPs (186, 259–261). These *in vitro* studies, although not on fully asymmetric membranes (236), broadly validate the observations of the above *in silico* studies.

Measurement of water permeation in *P. aeruginosa* PAO1 LPS (outer)–DPPE (*di*C_16:0_PE) (inner), *P. aeruginosa* LPS (outer)–PPPE (C_16:0_C_16:1_PE)/PVPG (C_16:0_C_18:1_PG)/CL (inner), and *E. coli* LPS (outer)–PPPE/PVPG/CL (inner) asymmetric bilayers by A-MD showed that the outer leaflet is relatively permeable to water when compared with the inner leaflet (with water reaching the terminal methyl groups of lipid A acyl chains) with both “rough” LPS and LPS containing an O-antigen region (246, 248,249, 262). This polarity gradient is also apparent through interactions between loop regions of OMPs and charged and polar groups on LPS that have been observed by A-MD and CG-MD (79, 210, 247–249, 253, 263–274) and specific LPS-binding sites that have been validated experimentally for trimeric porins such as OmpF (6, 189, 275–278). One area that remains understudied but warrants further investigation is the effect of the acyl tails of lipoproteins in the inner leaflet of the asymmetric OM on membrane properties. The copy number of lipoproteins at the IM is small enough to, presumably, have a negligible effect on the membrane's bulk physicochemical properties. However, at the OM there may be over 1 million lipoproteins anchored to the inner leaflet, composed primarily of Lpp (Braun's lipoprotein)—one of the most abundant proteins in *E. coli*—and Pal (3, 4, 279, 280). Each lipoprotein is triacylated at an N-terminal cysteine (121), and each acyl chain can be approximated to occupy ∼0.28 nm^2^ in a bilayer (100, 262,263, 281–285). Assuming a lower bound on the surface area of an average *E. coli* as 3.7 μm^2^ and an upper bound of 13 μm^2^ (286–288), and the total area occupied by lipoprotein tails similarly bounded between 0.25 μm^2^ (3 × 10^5^ proteins) and 1.1 μm^2^ (1.3 × 10^6^ proteins) (3, 4), we come to an estimate of 2–30% of the inner leaflet of the OM occupied by these lipid anchors. Lpp and Pal both bind peptidoglycan through their protein domains. This interaction restricts their mobility (and therefore the lateral mobility of their lipid anchors) and stiffens the OM, and the protein itself physically occludes the headgroups of the OM inner leaflet lipids (171, 289, 290). The impact that this has on the bilayer properties of the OM requires further study, but the presence of these lipoproteins would act to further reduce the “accessible” surface for OMPs to bind and initiate folding.

Just as early *in vitro* experiments on synthetic lipid vesicles allowed hypotheses to be generated about the properties of biological membranes, we can look to *in vitro* experimental models of asymmetric phospholipid membranes to infer information about how asymmetry may affect the OM. These systems remain experimentally challenging due to problems with mixing/scrambling of the inner and outer leaflets in both surface-deposited (291, 292) and liposome-based asymmetric membranes (293), as well as difficulties in accurately controlling the composition of the outer leaflet in asymmetric liposome-based studies (294, 295). Nonetheless, such studies have suggested that asymmetry results in coupling between leaflets, which alters the physical properties of the bilayer distinctly from those of the same lipid types mixed symmetrically. These include changes to the membrane potential difference, lateral pressure differential, and the packing of lipids (296–300).

Synthesizing the data described above from *in vitro* and *in silico* studies allows insights into the view of the membrane encountered by a nascent OMP as it approaches and is inserted into the OM. As the OMP approaches and moves through the membrane, it begins folding on a 'typical' liquid-disordered bilayer leaflet where phospholipids are free to diffuse before entering a region of low lateral mobility and increased hydration in the outer leaflet. These gradients of lateral mobility, lipid packing, hydration, and lipid headgroup polarity as an OMP inserts across the membrane normal could help to stabilize the tertiary structure of OMPs, particularly the hydrophilic loop regions (which can be >20 residues in length) (301), and drive the folding of the β-barrel domain to completion. Overall, therefore, the above studies have shown that the physical properties of the OM are highly complex and can vary dependent on the underlying lipid phase and the elastic stress, as well as the presence of OMPs and lipoproteins, and the LPR (175, 214, 302). These parameters, in turn, can be tuned by the lipids incorporated into each leaflet, especially by modifications to the LPS in the outer leaflet of the OM. Hence, bacteria have to be adaptable so that they can form and maintain their OM whatever the nature of their environment and the environmental stresses that they encounter.

Membrane-spanning OMPs need to insert through both leaflets of the OM to adopt their native, functional folds, yet how this unique asymmetry and changing lipid content of the OM affects the folding and function of OMPs is currently unknown. We now need to take a deeper look into what we know about OMP folding, starting with the basics and then building more complexity into experimental and theoretical models to allow us to understand how the unusual properties of the OM might influence the process of OMP biogenesis in bacteria.

## How do OMPs fold?

### 30 years of experiments on OMP folding in vitro

**Figure 6.**
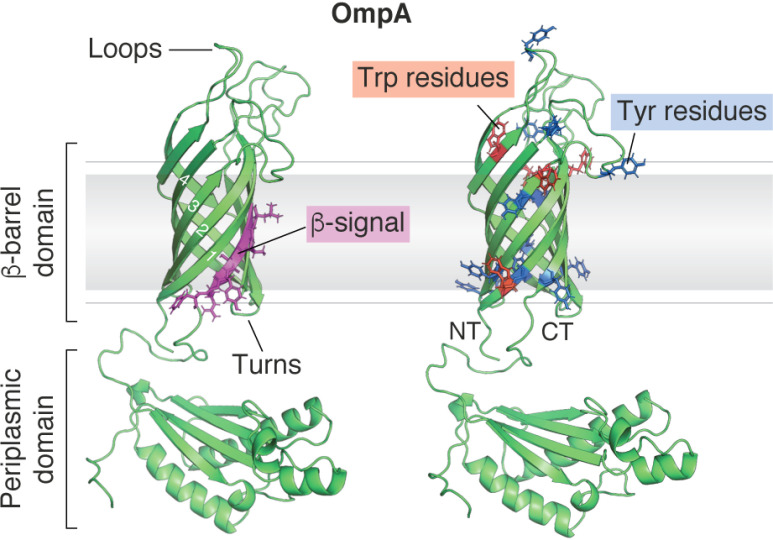
**The structure and architecture of OmpA.** The 8-stranded β-barrel OmpA has been used for many studies of OMP folding *in vitro*. It comprises a transmembrane β-barrel domain and a soluble periplasmic peptidoglycan-binding domain. Neighboring β-strands are connected on their extracellular side by a long disordered “loop” and on the periplasmic side by a short “turn.” OMP β-strands are usually numbered from the N terminus (*NT*) to the C terminus (*CT*), and the C-terminal β-strand often contains a conserved motif of Gly-*X-X*-Ar-*X*-Ar (where Ar represents any aromatic residue), indicated in *purple* on OmpA, thought to be important for recognition by BAM (386, 399). Many OMPs contain an enrichment in aromatic Trp and Tyr residues in their β-barrel domain, particularly at the interfacial region between the lipid headgroups (approximate position indicated by the *gray line*) and acyl tails (approximate position indicated by the *gray box*) known as an “aromatic girdle.” Trp (*red*) and Tyr (*blue*) residues found in the β-barrel domain of OmpA are indicated *above*. This model of OmpA was created in PyMol 2.X (Schrödinger, LLC) by fusing the NMR structures of the *E. coli* OmpA β-barrel (PDB code 1G90) (400) and its periplasmic domain (PDB code 2MQE) (401).

Likely due their high thermodynamic stability (Δ*G*°_F_ = −10 to −140 kJ mol^−1^) (303–312), relatively low hydrophobicity (by all-residue average on the Kyte–Doolittle scale most OMPs are hydrophilic due their alternating hydrophobic membrane facing/generally hydrophilic lumen facing patterning), and ease of recombinant expression and purification, OMPs are unusually tractable models for *in vitro* studies of membrane protein folding. The first published study on the successful folding of OMPs *in vitro* was carried out in 1978, when it was shown that SDS-boiled and denatured OmpA (Fig. 6) could be refolded with high yield into LPS (but not into solutions containing total *E. coli* phospholipids, DMPC (*di*C_14:0_PC), or the sugar moiety of LPS) (313). This refolded OmpA was shown to be natively structured because it had regained function (activity in phage receptor binding assays), was protected from protease digestion, and migrated at an anomalous molecular weight in SDS-polyacrylamide gels when loaded without boiling (cold SDS-PAGE) (313). Hence, the stage was set for detailed studies of the mechanisms of OMP folding, at least for this and other small (8-stranded) OMPs.

The next major breakthroughs in our understanding of OMP folding were made in the 1990s, when it was shown that OmpA and an unnamed porin could be spontaneously refolded *in vitro* into detergent (octyl glucoside), small unilamellar vesicles (SUVs) of DMPC (*di*C_14:0_PC), or mixed lipid-detergent micelles (octyl-poly-oxyethyleneoxide (C8POE) and soybean lecithin) in the presence of SDS without the addition of an external energy source or other proteins (314–316). Soon after, several groups began to experiment with the refolding conditions to identify the determinants that allow OMPs to attain their native structure. These studies showed that detergents are required to be above their critical micelle concentration for successful OMP folding (317, 318). This highlighted the importance of a surface to initiate folding and showed that simple binding of hydrophobic molecules around an unfolded OMP chain is insufficient to enable folding. Instead, some degree of preorganization is required. These studies also showed that folding of OMPs *in vitro* is a remarkably slow process (most OMPs taking on the order of minutes to hours to fold), too slow to be physiologically relevant (the doubling time of *E. coli* is ∼20 min at 37 °C). They also showed that refolding yields are improved by the addition of urea, implying that the OMP-folding landscape contains kinetic traps and/or off-pathway intermediates or aggregates that can be suppressed by the addition of chaotrope (317, 319). These experiments also showed that the β-barrel transmembrane fold is extremely stable once formed, with native OMPs being resilient to denaturation by SDS (unless heated to high temperatures), enabling kinetic measurement of folding using cold SDS-PAGE (71, 313, 320). This is due to the high kinetic barrier to unfolding in SDS (on the order of years at 30 °C for OmpA) (314, 321).

Due to their high kinetic and thermodynamic stability, high concentrations of denaturant often fail to unfold OMPs that have been refolded into detergent micelles or lipid bilayers. For example, OmPLA remains enzymatically active in 8 m urea and in 6 m guanidine-HCl (317). Experiments measuring the activity of OmPLA following refolding into LPS and a range of detergents showed not only that the protein can reacquire a stable fold, but also that it regained its phospholipase activity, confirming reversible folding to a functional state (317). Thus, OMPs obey Anfinsen's dogma that all of the information for a protein to reach its thermodynamically stable native structure is contained in its amino acid sequence (322).

### First glimpses of an OMP-folding mechanism

**Figure 7.**
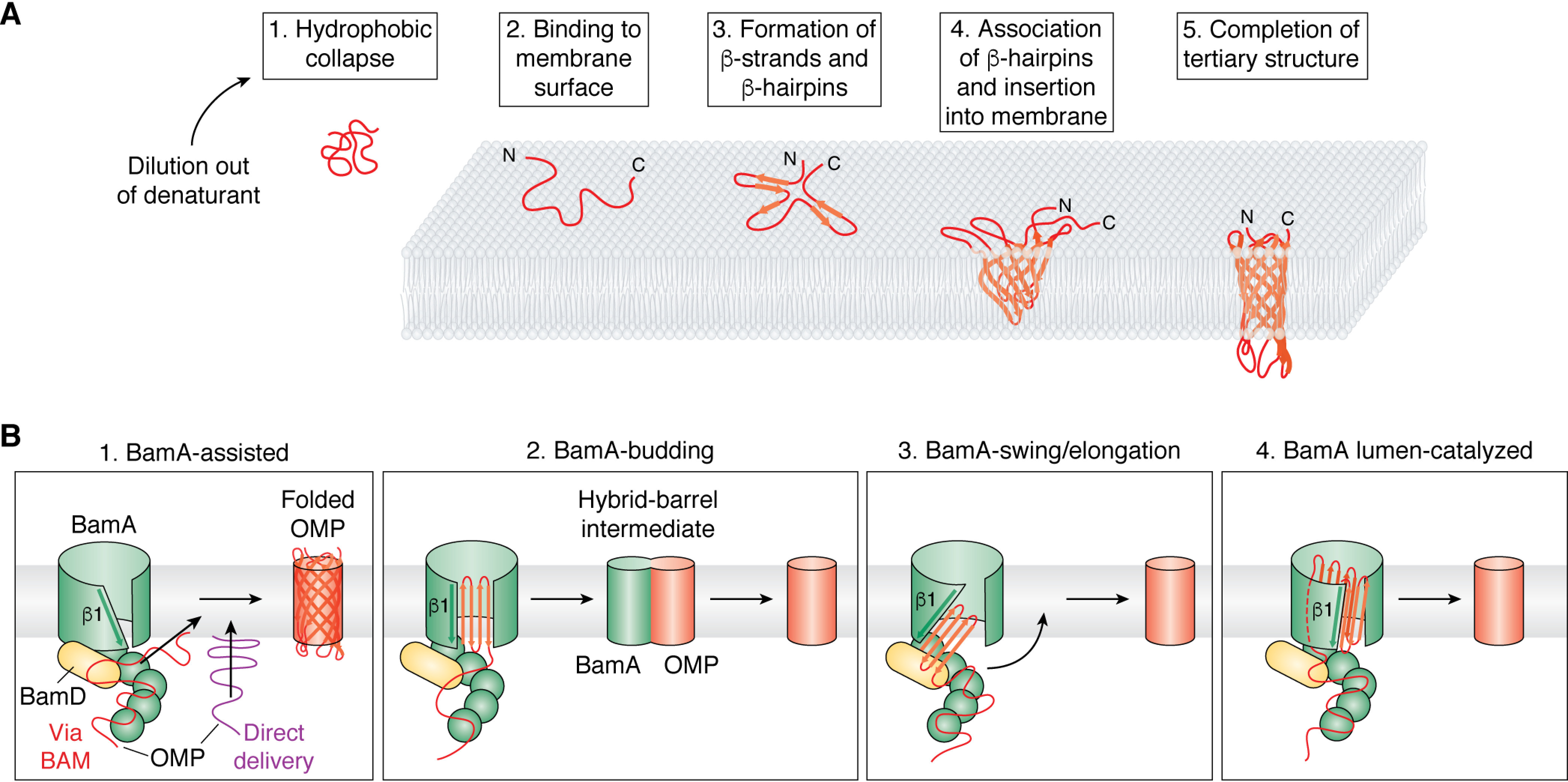
**Mechanisms of OMP folding *in vitro* and *in vivo*.** *A*, Mechanism of spontaneous OMP folding *in vitro* as described for OmpA. *In vitro* studies have shed light on the folding pathway of model OMPs, particularly OmpA, after dilution out of high concentrations of denaturant in the presence of a lipid bilayer. Adapted from Danoff and Fleming (351). This research was originally published in Biochemistry. Danoff, E. J., and Fleming, K. G. Membrane defects accelerate outer membrane β-barrel protein folding. *Biochemistry* 2015; 54:97–99. © American Chemical Society. *1*, immediately after dilution out of denaturant, the chain undergoes hydrophobic collapse; *2*, the polypeptide chain then binds to the surface of a membrane; *3*, the nascent OMP then begins to form secondary structure as it brings together neighboring β-strands to form β-hairpins while still mostly exposed to the aqueous environment; *4*, these β-hairpins associate and begin to insert into the acyl tail region of the membrane; *5*, the tertiary structure of the barrel is complete with the final step likely being a slower equilibration of side chains and extrusion of hydrophilic loops from the barrel lumen. *B*, proposed mechanisms of BAM-catalyzed folding of OMPs *in vivo*. The nascent OMP is shown in *red* or *purple*, BamA is shown in *green*, and BamD is shown in *yellow* (other subunits have been omitted for clarity). *1, BamA-assisted*. Substrate OMPs are delivered to BAM or directly to the membrane by periplasmic chaperones. These nascent OMPs then fold spontaneously into a region of destabilized membrane in front of the lateral gate of BamA, essentially following the same pathway as described in *A*. *2, BamA-budding*. Binding/recognition of the OMP occurs on BAM before β-strands are added a β-hairpin at a time between β1 and β16 of BamA, forming a semisymmetric hybrid-barrel intermediate. Once all β-hairpins are added, this folded OMP then buds off from BamA. *3, BamA-swing/elongation*. Binding/recognition of the OMP occurs on BAM and folding starts with templating of the C-terminal β-strand of a nascent OMP against β1 of BamA. Folding proceeds in the periplasm through the stepwise addition of more β-strands. Once all β-strands have been added, a conformational change in BamA “swings” the folded β-barrel into the membrane. *4*, *BamA lumen–catalyzed*. This model begins as described in *3* with templating against BamA β1. However, formation of further β-strands is catalyzed against the lumen wall of BamA with a conserved motif in loop 6 of BamA (not shown) possibly stabilizing this interaction. In all of these models, BamD (*yellow*) may play an important role in substrate recognition and/or the conformational cycle.

The next era of work on OMP folding focused on attempts to determine the mechanisms of folding, including why the process is so slow, whether partially folded intermediates are formed, the nature of folding transition states, and the role of the protein sequence, lipid composition, and folding factors (including BAM and molecular chaperones) in the folding process. In some of the earliest studies of this kind, Surrey *et al.* (316) measured the kinetics of OmpA folding into SUVs formed from 95% DMPC (*di*C_14:0_PC), 5% DMPG (*di*C_14:0_PG) (mol/mol) at a temperature above the lipid *T_m_* by rapid dilution from urea *in vitro*. The techniques employed and optimized in these early studies of OMP folding form the toolset used for such studies to this day (323). They also reinforced the idea garnered from studies on the folding of water-soluble proteins (324–327) that insights into the mechanisms of folding can be best learned by taking a kinetic approach to dissect each step in the folding pathway. These methods include (i) monitoring the change in tryptophan (and/or tyrosine) fluorescence as an OMP folds from aqueous solution into a nonpolar membrane (Fig. 6 shows the location of aromatic residues on OmpA); (ii) measuring the formation of secondary structure using far-UV CD, and (iii) following the formation of SDS-resistant molecules (presumably containing a correctly folded β-barrel domain) using cold SDS-PAGE (316, 328). This work showed that OmpA folds into lipid bilayers *in vitro* via a multiphasic mechanism involving rapid hydrophobic collapse that occurs in the experimental dead time (∼1 s), followed by two slower phases occurring in minutes (phase 1) and tens of minutes (phase 2) corresponding to structural rearrangement and concurrent formation of secondary and tertiary structure (Fig. 7*A*) (316). It was also shown that pH affects the yield and rate of OMP folding, with pH values close to the pI of OmpA (pI ∼5.7) increasing the folding rate but decreasing the folding yield. The average pI of the OMPs shown in Fig. 1 is ∼5.4, and the environment in which *E. coli* has evolved (the large colon) is mildly acidic (pH 5.5-7.5) (329), suggesting that *E. coli* has adapted to favor rapid folding and has systems to handle or circumvent the lower yields. Higher LPRs were also shown to increase the folding rate and yield, consistent with models suggesting that the OMP first binds to the surface of the lipid bilayer before folding and insertion are completed (316, 330–332). Indeed, refolding studies of OmpA into lipids above their *T_m_* confirmed this membrane-binding step with elegant experiments using membranes composed of lipids that had been brominated at different positions along the acyl chain (bromination quenches fluorescence from tryptophan only when in close proximity, thereby allowing depth-dependent changes in fluorescence) (333, 334). Earlier data from ATR-FTIR studies showed that this membrane-adsorbed (but not stably integrated) folding intermediate had significant β-sheet content, with β-strands that have still to adopt the orientation found in the native β-barrel (335). Finally, the finding that tryptophan residues located in β-strands, near extracellular loops, or in intracellular turns show similar rates of membrane insertion (judged by tryptophan fluorescence quenching) suggested that folding occurs via a concerted mechanism in which all four hairpins of this 8-stranded OMP move synchronously across the bilayer (330), increasing in tilt angle as the protein becomes fully membrane embedded (335). More recent experiments employing single-Cys single-Trp variants of OmpA labeled at the Cys with Trp-quenching nitroxyl spin labels have shown that despite being concerted, OmpA's β-strands form in a particular order (336), with β-strands initially associating with each other at their “extracellular” sides like a tipi (*i.e.* before the “periplasmic” side forms) (Fig. 7*A*, step 4) and showing that the N- and C-terminal strands are already in close proximity in the membrane-adsorbed state (Fig. 7*A*, step 3), providing evidence against models invoking β-barrel closure as the last step (336). These experiments also failed to detect native β-strand association in aqueous solution, ruling out models in which the OmpA β-barrel “pre-folds” in solution before inserting as a single unit into the membrane. This complex, multistep process where formation of secondary and tertiary structure is coordinated contrasts markedly with the two-stage mechanism proposed for α-helical membrane proteins in which the formation of α-helices precedes formation of the native tertiary structure (337, 338). The overall general model for spontaneous OMP folding *in vitro* is summarized in Fig. 7*A*.

### The role of the membrane in OMP folding

Studies varying the lipid headgroup, acyl chain length, lipid phase, membrane curvature, and elastic curvature stress (the change in lateral pressure across the membrane) (Fig. 5) have shown that the properties of the membrane can have dramatic effects on the folding of OMPs. For example, whereas OMPs such as OmpA and PagP (both 8-stranded; Fig. 1) are able to fold into highly curved SUVs comprised of DOPC (*di*C_18:1_PC) or DMPC (*di*C_14:0_PC) in minutes, folding is slower or effectively prevented (beyond the timescale employed in the study) when these proteins are folding into LUVs formed from DOPC (*di*C_18:1_PC) under the same experimental conditions (40). Similar studies have recapitulated these results and shown that the elastic curvature stress, hydrophobic mismatch (hydrophobic thickness of the OMP *versus* the bilayer), and membrane curvature (SUV *versus* LUV) affect OMP folding, emphasizing the importance of the physical properties of the lipid environment in determining the folding process (339, 340). The steep decrease in refolding rates observed as the acyl chain length of PC lipids increases (330) and the absence of reports of spontaneous folding into LUVs formed from pure PC lipids with saturated acyl chain lengths >14 carbon units (*i.e.* >DMPC (*di*C_14:0_PC)) can be rationalized by the elastic free energy of the membrane, as this parameter is expected to increase to the fourth power of membrane thickness (341). Membrane thickness is also thought to relate to the incidence of packing defects and thermal fluctuations in the bilayer, with thinner membranes having more defects. Such packing defects may be responsible for the more rapid folding of OMPs into thinner bilayers (341). OMPs have also been shown to be unable to fold into some lipids, such as DMPC (*di*C_14:0_PC) and DPPC (*di*C_16:0_PC), in the gel phase, but they fold readily when the same lipids are in the liquid-disordered phase, suggesting a significant activation energy barrier for the protein to insert across the bilayer (333, 334). Accordingly, the folding rate and yield of tOmpA (the β-barrel domain of OmpA) and OmpX (8-stranded; Fig. 1) into micelles (or mixed micelles of different lipid/detergent types) can be increased 10−100-fold by applying a transient “heat shock” (∼70 °C) during folding (342). This heat shock presumably confers enough thermal energy to rapidly take the unfolded ensemble over the activation energy barrier. It has also been shown that whereas gel-phase lipids (below their *T_m_*) prevent or retard folding, and more rapid folding is observed into lipids in their liquid-disordered phase, folding is most rapid at the interface between these phases (*i.e.* at the *T_m_*) (343). At this temperature, regions of gel-phase (ordered acyl chains) and liquid-phase (disordered acyl chains) lipids coexist (344, 345), and frustration between the packing requirements of these phases is believed to generate packing defects (as shown by an increase in solute permeability) (346–350), which may be responsible for the acceleration of OMP folding. Kinetic modeling experiments have suggested that this phenomenon involves an acceleration in the formation of an early membrane-inserted folding intermediate (351).

Together, these data have established the importance of the chemical and physical properties of the membrane environment (lipid headgroup, acyl chain length, size, and curvature of the membrane vesicle) and the intimate connection between the protein sequence and membrane into which the OMP must fold.

### Commonalities in the folding mechanisms of OMPs in vitro

OMP-folding intermediates formed by initial adsorption to the lipid bilayer surface have been observed for several small OMPs, including the 8-stranded OmpA (see above) and PagP (332). An extensive mutational study of the folding kinetics and equilibrium stability of single point mutants of PagP revealed the first evidence for the nature of an OMP-folding transition state (in DLPC (*di*C_12:0_PC) LUVs), revealing that this species, which is formed subsequent to initial folding on the membrane surface, contains a partially folded β-barrel, in which the C-terminal strands have formed native-like contacts, but the N-terminal strands remain largely unstructured (352). The results from this study also suggested that PagP is tilted in the membrane in the transition state ensemble, reminiscent of CG-MD studies observing the insertion of preformed OmpA into a DPPC (*di*C_16:0_PC) bilayer (353). Kinetic analysis of PagP folding also showed that this OMP folds via parallel pathways, with the route taken depending on the nature of the lipid employed (332). Similar results showing parallel folding pathways were also obtained for FomA (331), yet again highlighting the importance of the membrane surface and the physical state of the lipid bilayer for OMPs to fold (331, 332).

The view of OMP folding *in vitro* that emerges from these studies shows a common first step involving folding on the lipid surface followed by preorganization of the approximate structure of the OMP, with translocating aromatic residues anchoring the nascent β-barrel to the membrane, and association of neighboring β-strands causing a hydrophobic surface to be displayed toward the membrane. This would, in turn, drive the energetically favorable partitioning of the hydrophobic protein surface deeper into the acyl chains (354). At the same time, hydrogen bonding between β-strands during insertion may be driven by the energetic penalty associated with displaying the unbonded polar peptide backbone to the nonpolar membrane environment. Finally, the association of β-strands in the correct order may be a rate-limiting step for insertion into the bilayer.

## BAM: Nature's answer to the challenges of OMP folding

Whereas many OMPs can be folded *in vitro* into SUVs and LUVs composed of short chain lipids, attempts to fold these proteins into liposomes comprised of *E. coli* polar lipid extract result in moderate folding yields for some OMPs (OmpA (8 strands), OmpT (10 strands), and BamA (16 strands)) and poor or no folding for others (OmpX (8 strands), PagP (8 strands), OmpW (8 strands), OmPLA (12 strands), and FadL (14 strands)) (Fig. 1) (40, 41). Spontaneous folding of OMPs of many sizes has been observed into DDPC (*di*C_10:0_PC) LUVs (40) but has been shown to be suppressed upon incorporation of lipids with headgroups native to the *E. coli* OM that confer negative spontaneous curvature (PE) or net negative charge (PG) (41). BamA is a 16-stranded essential OMP (Fig. 1) and is the most conserved subunit of the multiprotein BAM complex, which is involved in the biogenesis of other OMPs. BamA or a BamA variant lacking most (4 of 5) of its periplasmic polypeptide transport-associated (POTRA) domains can partially rescue the poor folding efficiency of OMPs into these liposomes (41). These results highlight the vital role of BAM for OMP folding into lipids commensurate with those found *in vivo*, suggesting that spontaneous folding of OMPs into a native lipid bilayer *in vivo* is kinetically unfavorable unless BAM is present.

### BAM as a modulator of the physical properties of membranes

**Figure 8.**
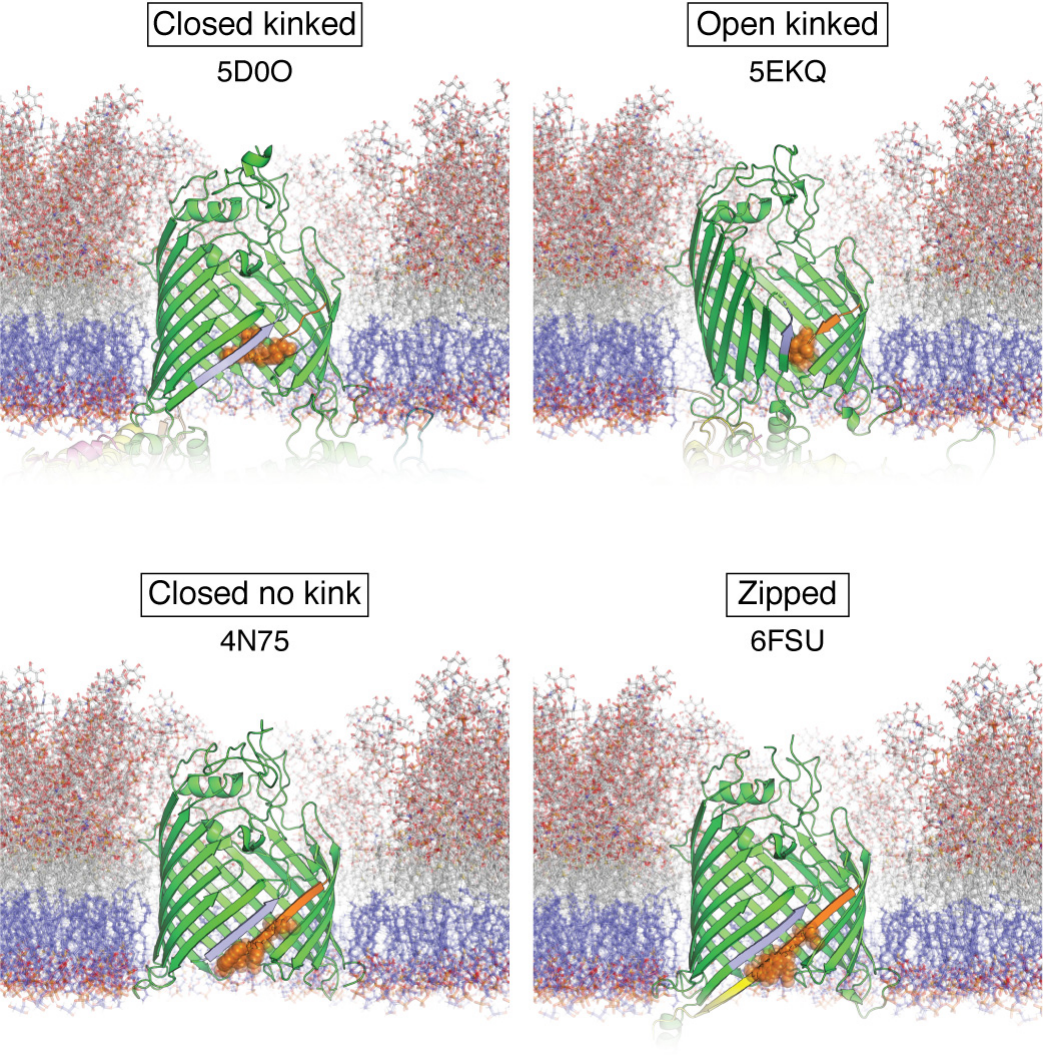
**Conformations of the BamA lipid-facing lateral gate.** Shown are example structures of *E. coli* BamA adopting different conformations around the location of the β1–β16 seam/gate. BamA has been observed in both gate open and closed states with the open state observed in the presence of other BAM subunits but not in structures of BamA in isolation. Furthermore, in all structures of the full BAM complex, β16 of BamA adopts a kinked conformation at a highly conserved glycine (Gly^807^) in both the open (PDB code 5EKQ; BamACDE) (368) and closed (PDB code 5D0O; BamABCDE) (357) states of the gate. Residues Asn^427^–Gly^433^ (β1) are *highlighted* in *light blue*, residues Phe^802^–Trp^810^ (β16) are indicated in *orange*, and the kink is further highlighted with *spheres* (Ile^806^–Trp^810^). This kink is also observed in structures of BamA from *Salmonella enterica* (PDB code 5OR1) (370) and *Neisseria gonorrhoeae* (PDB code 4K3B) (355) (not shown) and in the BamA homologue, TamA, which also plays a role in OMP assembly (PDB codes 4N74 and 4C00) (387) (not shown). BamA with a closed gate and no kink has been observed in isolation (PDB code 4N75; BamAΔ1–427) (366) and in a hybrid BamA containing a C-terminal 9-residue extension (*colored yellow*) comprised of part of turn 3 and β7 from OmpX, which may represent a mimic of an OMP-BamA folding intermediate (PDB code 6FSU) (371). Structures are represented in an asymmetric bilayer with a mixture of phospholipids with 14-18 carbon acyl chains (shown in *violet*) in the inner leaflet and *E. coli* rough LPS in the outer leaflet (acyl chains in *white*). Note the different hydrophobic thickness between each leaflet. Asymmetric bilayer was built using the GNOMM server (402).

Until recently, how BAM folds OMPs remained mysterious. The solution of several structures of BamA and BAM, as well as kinetic experiments, are now starting to provide glimpses of how this amazing machinery functions and how it might involve remodeling of the lipid bilayer. MD simulations of BamA and the full BAM complex have shown that the presence of the 16-stranded BamA β-barrel causes thinning and disordering of the membrane in the vicinity of its β1–β16 seam and that BamA can switch between conformations at this interface (355–360) (Fig. 8). Other OMPs have been shown to generate variable (*i.e.* anisotropic) membrane thickness around their circumference experimentally (*e.g.* BtuB, 22-stranded) (361), and more isotropic bilayer thinning is seen in A-MD and CG-MD simulations in the vicinity of many OMPs (OprH (8-stranded from *P. aeruginosa*), OmpA, LpxR (12-stranded from *S. typhimurium*), Hia (3 × 4 strands from *Hemophilus influenzae*), OmPLA, NanC, OmpF, LamB, and FhuA) (186, 209, 248, 249, 267, 274, 359, 362). Although the dynamics of lipids surrounding BamA have not yet been studied *in vitro* or *in vivo* (*e.g.* by using spin-labeled lipids), experiments examining the lipids directly surrounding other OMPs have found them to be motionally restricted in the vicinity of the β-barrel, as seen for FomA (8-stranded from *Fusobacterium nucleatum*), OmpA, OmpG, and FhuA (363–365). This has also been observed in CG-MD simulations of OmpA, NanC, OmpF, LamB, and FhuA in a bilayer formed from 75% POPE (C_16:0_C_18:1_PE), 25% POPG (C_16:0_C_18:1_PG) (186). It should be noted, however, that it is not certain that the two parameters of lipid order and motional restriction are always correlated—consider a situation where disordered lipids are corralled by ordered lipids. With current data, it is unclear whether the lipid disordering observed around the β1–β16 seam of BamA is purely a consequence of membrane thinning (*i.e.* a large hydrophobic mismatch) or is accentuated by some other mechanism in this OMP. Evidence for such a reaction cycle that could support a membrane-remodeling mechanism for BamA was suggested from crystal structures of BamA in isolation and from crystal and cryo-EM structures of BamA in the BAM complex. These studies showed that the BamA β-barrel is shorter in the β1–β16 region than in the rest of the barrel and that it can explore at least three distinct and potentially membrane-influencing conformations: a closed fully zipped (*i.e.* hydrogen-bonded along its whole seam) barrel (Fig. 8, closed no kink and zipped), a closed partially zipped barrel (Fig. 8, closed kink), and an open barrel (Fig. 8, open kinked) (42, 91, 355, 357, 366–371). This open conformation was surprising, as it would intuitively seem highly energetically unfavorable to break hydrogen bonds in a hydrophobic environment. However, WT BamA is only capable of forming at most 6-8 backbone hydrogen bonds between its first and last β-strands (Fig. 8, closed no kink). MD simulations showed that this fully hydrogen-bonded conformation is unstable and eventually forms the “partially zipped/closed kink” conformation seen in structures of the full BAM complex (Fig. 8, closed kink) where the terminal strand bends back into the barrel lumen, leaving just 2–3 hydrogen bonds (360). This partially zipped state may lower the energetic cost of fully opening the BamA barrel (360), leaving at most one hydrogen bond between β1 and the periplasmic turn between β15 and β16. This periplasmic turn is likely to be important in stabilizing this open state, as its mutation or truncation is lethal *in vivo* (370). This still requires the breaking of 5–7 hydrogen bonds, but some of this may be compensated by the kinked residues hydrogen-bonding to water within the lumen of the BamA barrel. Furthermore, the cost for OMPs to break hydrogen bonds in the membrane may be lower than previously assumed (372), and the outer leaflet of the OM may be more hydrated than a symmetric phospholipid bilayer. These effects could help stabilize the structure of BamA while effecting local changes in packing or stability of the membrane. The importance of this BamA barrel opening was highlighted by the lethality of disulfide bonds engineered to lock the barrel closed (356) or open (357). Interestingly, folding of OmpT and OmpX via BamA or the full BAM complex *in vitro* is still catalyzed in the locked closed state (42, 373). This implies that the structure of BamA alone, with its reduced hydrophobic thickness around β1–β16, may intrinsically accelerate OMP folding by distorting the membrane—even in the absence of the open state. *In vitro*, BamA has been shown to have a greater catalytic effect on tOmpA folding (higher catalytic fold rate enhancement) as the hydrophobic thickness of a bilayer is increased from ∼19.5 Å in DLPC (*di*C_12:0_PC) LUVs to ∼23.0 Å in DMPC (*di*C_14:0_PC) LUVs, showing that hydrophobic mismatch and/or lipid disordering plays an important role in the mechanism of BamA-assisted folding (359). These data illustrate how both structural and biochemical approaches will be required to fully understand the function of the BAM complex.

Recent studies have also shown the importance of BAM and membrane fluidity in folding OMPs *in vivo.* These studies exploited a mAb that was found to be bactericidal, binding to extracellular loop 6 of BamA (93). Interestingly, bacteria showing spontaneous resistance to this BAM-mediated toxicity were found to have mutations in the *lpxM* gene. This protein transfers a C14 (myristoyl) chain to penta-acylated LPS, creating hexa-acylated LPS (93). Antibody sensitivity was restored (*i.e.* bactericidal effects of the antibody were reinstated) when *lpxM* was expressed from a plasmid. Assays of membrane fluidity using a pyrene-based probe showed that membrane fluidity decreased in the resistant strains, and this effect was recapitulated in other conditions that decrease membrane fluidity (high salt, longer LPS sugar region, lower temperature) (80, 93). The levels of OMPs were not reduced in Δ*lpxM* strains in the absence of the antibody. This suggests that there is a mechanistic link between BamA/BAM and membrane fluidity, as BAM is most sensitive to inhibition when the membrane is more fluid. Hence BamA activity (as part of the BAM complex) may be lower when the OM is excessively fluid, confirming a link between OMP-folding efficiency, BAM activity, and membrane fluidity *in vivo.* More generally, it is interesting to consider the role of the lipids that directly surround BAM and how they might interact with it. Most MD studies on BamA have utilized simple lipid mixtures (355–360). However, it is known that membrane proteins can enrich lipids or other proteins around them that match their hydrophobic thickness (374–376). In the context of BAM, this may have two consequences. First, shorter-chain lipids may be enriched around the β1–β16 seam to reduce the energetic penalty of membrane thinning, with C_12:0_ and C_14:0_ acyl tails found in *E. coli* and also reported to be more abundant in the OM than the IM (139). Second, the hydrophobic thickness of substrate OMPs is generally greater than that of BamA. This means immediately after insertion into the region surrounding the BamA β1–β16 seam, the newly folded or folding OMPs will encounter a region of positive hydrophobic mismatch (Fig. 5), and it may be energetically favorable for them to diffuse away to regions of greater hydrophobic thickness with a more optimal match to their size (376). This could provide a mechanism for release and local clearance of newly folded OMPs from BAM. Furthermore, although substrate binding to BAM subunits or the POTRA domains of BamA is likely to be important in conformational cycling (60, 377–382), the lateral pressure or fluidity (Fig. 5) within the OM might also play a role in controlling opening and closing of the BamA barrel. These dynamics are thought to be essential for its role in catalyzing OMP folding *in vivo* (356, 383), so modulation of the BamA barrel dynamics by the lipid environment may also provide a secondary mechanism of controlling the function of BAM.

### A multifaceted mechanism for the BAM complex

Growing evidence points to a mechanism of BAM function in catalyzing OMP folding and membrane insertion that involves the templating of C-terminal β-strands of the nascent substrate OMPs onto the β1 strand of BamA (Figs. 6–8) (47, 61, 384). The C-terminal strands of OMPs in bacteria and mitochondria contain a conserved aromatic-rich motif termed the β-signal (Fig. 6), which may be important in the recognition of OMP clients by the BAM complex (385, 386). The initial recognition of the OMP's C-terminal strand is thought to then trigger nucleation of further β-hairpins to the growing β-barrel, perhaps favored by the reduced entropic penalty of folding (as the degrees of freedom of the unfolded chain are reduced). However, the steps following this initial binding event, particularly how the β-barrel of the newly forming OMP folds and inserts into the membrane, remain unclear. A role for the lateral gate of BamA in this process was proposed after the first structures of this family of proteins were solved (355, 387, 388). Further experimental evidence suggests four possible models for how this is achieved (Fig. 7*B*). 1) BamA plays a passive role in OMP folding/insertion and merely targets nascent OMPs to a destabilized bilayer in front of its β1–β16 seam. Substrate binding possibly initiates a conformational change in BamA, increasing its “lipid disorderase” activity, but folding otherwise proceeds as in the *in vitro* pathway (Fig. 7*A*) (the assisted model) (388). 2) The β-barrel grows laterally into the membrane after templating onto the BamA gate (the budding model) (384, 388). 3) Prefolding/elongation of the OMP occurs in the periplasm after binding to β1 of BamA. A conformational change in BAM then inserts the already-folded β-barrel (the swing/elongation models) (47, 389). 4) Substrates fold against the interior wall of BamA while keeping their N and C termini in close proximity ready for β-barrel closure and release into the membrane (the lumen-catalyzed model) (61). The last two models are particularly intriguing as they suggest that the hydrophobic surface of the folding OMP is partially exposed to an aqueous or polar environment. How this step could be energetically favorable is not yet clear, but some authors have proposed that the cradle created by the BamA lumen and POTRA domains may aid folding by acting like an entropic cage, analogous to chaperonins such as GroEL/ES (61, 389). This may drive folding by reducing the conformational entropy of the unfolded state but may also restrict the mobility of water, perhaps offsetting some of the cost of exposing hydrophobic residues.

Through its mechanism of β-strand capture, the BAM complex may also act to suppress the reversible off-pathway intermediates in OMP folding that cause kinetic retardation of folding *in vitro* and which may be related to both misfolded monomeric intermediates and aberrant transient intermolecular interactions (351). Furthermore, these aberrant multimers (so called “elusive” states as they are not easily observed by SDS-PAGE) are not observed in the absence of a lipid bilayer (351), implicating the OMP-membrane interaction as an important control point where these states could form. The nature of the terminal stages of folding, including how the OMP is able to partition rapidly into the OM and how it overcomes the activation energy barrier associated with membrane insertion, remain unresolved. Finally, the enrichment of shorter-chain phospholipids and depletion of OMPs around BamA proposed above would essentially “clear some space” for folding to proceed (*e.g.* see Fig. 3*C*), providing a mechanism that could overcome the remarkably low LPR of the OM and an inner leaflet crowded with OMPs and lipoproteins. The proposed formation of “supercomplexes” that span the periplasm linking the IM SecYEG translocon and the BAM complex at the OM could also provide a direct conduit for OMP biogenesis (390–392). If only limited patches of free lipid exist, it would be important to direct OMPs to these regions of the OM before they misfold or aggregate.

## Comparison of folding *in vitro* and *in vivo*

Our current knowledge about the physical constraints of OMP folding into lipid bilayers, the properties of the OM, the folding of OMPs *in vitro*, and the mechanism of BAM action all point to the membrane as an important interface for preorganization of OMPs into an insertion-competent state. Despite clear evidence for the importance of this early folding step, the unusually low LPR of the OM of *E. coli* (which may be as low as 6–14:1) (139, 156, 157) calls into question the direct interaction of the OMP and the OM during folding *in vivo*. Given this dearth of lipid surface and the observation that the rate of OMP folding falls dramatically with decreasing LPR (316, 331, 332), how do OMPs insert into the OM on a biologically meaningful timescale? Large lipid-rich patches could exist in the OM, but *in vivo* imaging studies have shown that BAM and newly inserted OMPs appear together in protein-rich clusters (56, 149, 181). This means that there are either very few free lipids (Fig. 3*A*) or only small, local, lipid-enriched domains (Fig. 3*C*). Hence, BAM may have evolved to provide an initial nucleation site for OMP folding in a generally lipid-poor environment and to accelerate this process, particularly for rapidly growing organisms such as *E. coli*. This would also be necessary to pack the OM to the high protein density that is observed *in vivo*. Although the recognition of nascent OMPs and the nucleation of β-barrel formation through templating/recognition of the newly folding OMP's β-strands on BamA would provide a rate enhancement in folding, the dramatic differences of folding rates into detergent micelles (fast) *versus* LUVs (slow) suggest that the ability of an OMP to navigate its folding pathway to find the native fold is not necessarily a major rate-limiting factor. Instead, it appears that membrane insertion and disruption of lipid packing could play a more significant role in controlling the rate of folding. *In vitro* experiments have shown that the greater the lateral pressure, lipid order, and packing of lipids within a bilayer, the greater the activation energy barrier for folding (41, 339, 340). However, local or transient defects in the packing of lipids in a membrane can allow OMPs to bypass a slower folding pathway and instead fold more rapidly (as shown by studies on the propensity of thinner bilayers and lipids at their *T_m_* to accelerate OMP folding) (40, 343). The physical structure of the OM remains poorly defined, but what we know is this: already-folded OMPs are resistant to deformation in the OM, LPS can rigidify the outer leaflet, and *in vivo* measurements suggest that at least some proportion of lipids in the OM are relatively ordered and less mobile. BAM may introduce local defects into this environment (by thinning of the membrane and causing local disorder of the lipids) to lower the energy barrier to insertion and thus accelerate OMP folding into this otherwise impenetrable membrane barrier.

## Concluding remarks and open questions

The OMP-folding problem *in vivo* can be simplified as the interplay between three factors: enzyme (BAM), substrate (OMP), and solvent (lipid). To fully understand OMP folding *in vivo*, it is necessary to characterize the relationships between these three dominating factors to understand their holistic function. BAM acts to catalyze the folding of OMPs into a lipid bilayer, and it may do this by nucleating the folding of the unfolded OMP substrate (BAM-OMP interaction) and/or by locally disrupting the packing of lipids (BAM-lipid interaction) to lower the activation energy of folding (OMP-lipid interaction). The evidence for the action of BAM on lipids is currently restricted to *in silico* experiments, although we can imply its importance from *in vitro* studies of the effect of the physical and chemical properties of the bilayer on OMP folding. More studies are now needed to determine the relative importance of this “disorderase” activity of BAM in ensuring that OMPs can gain access to the highly crowded and lipid-poor OM.

Our current lack of understanding of the physical properties of the OM prevents the generation of accurate models for the mechanism(s) of BAM in the OM, the effect of BAM on the structure and dynamics of the OM lipids (and vice versa), and how these changes impact OMP folding. Although levels of OMPs fall upon depletion of BAM *in vivo*, and OMP insertion kinetics are slowed in the absence of BAM *in vitro*, which OMPs require BAM for folding into the OM *in vivo* is still not known unequivocally. Indeed, some OMPs have been shown to be capable of folding without the aid of BAM, and others are aided by other proteins such as those of the localization of lipoproteins (Lol) machinery (Table 1). The asymmetry of the OM, combined with the mixture of lipid types, high fraction of proteins, and tethering to the peptidoglycan layer, makes the journey taken by a nascent OMP to the OM and its insertion *in vivo* an immensely challenging task to replicate *in vitro*. Future *in vitro* work should focus on understanding the OM and OMP folding in a context closer to that found *in vivo* and also on better understanding the effects of suppressor variants on the catalytic activity of BAM, so as to narrow the gap between *in vivo* and *in vitro* insights. At the same time, modern biophysical and biochemical tools are also needed to make these same measurements directly on OMPs in their native context within bacteria. *En route* to this, the use of outer membrane vesicles directly derived from bacteria may provide an excellent stepping stone (393–395).

In summary, despite enormous progress in our understanding of how OMPs fold *in vitro* and in dissecting the interactions and mechanisms of BAM, we still do not fully understand how BAM folds OMPs of different size and sequence; nor do we understand fully the role of the asymmetric OM lipid bilayer in defining this process. OMP folding has moved in the last few years from a question of fundamental importance and interest to one having direct implications for the development of new antibiotics targeting OM biogenesis. The challenge has thus been set to define how OMPs fold *in vivo* and to solve the remaining mysteries, so that BAM can be targeted to break the OM barrier and render pathogenic bacteria susceptible to attack by new antimicrobial agents.

## Note added in proof

After this manuscript was accepted for publication, a cryo-EM structure of a substrate-engaged BAM complex purified after stalling *in vivo* was reported supported by *in vivo* cross-linking. This structure shows a late-stage assembly intermediate and, along with the cross-linking data, appears to support model 3 (BamA-swing/elongation) or 4 (BamA lumen-catalyzed) of BAM-catalyzed OMP folding shown in Fig. 7 (403).
